# A classification based framework for quantitative description of large-scale microarray data

**DOI:** 10.1186/gb-2006-7-4-r32

**Published:** 2006-04-20

**Authors:** Dipen P Sangurdekar, Friedrich Srienc, Arkady B Khodursky

**Affiliations:** 1 Department of Chemical Engineering and Materials Science, University of Minnesota, Saint Paul, MN 55108, USA; 2 Biotechnology Institute, University of Minnesota, Saint Paul, MN 55108, USA; 3 Department of Biochemistry, Molecular Biology and Biophysics, University of Minnesota, Saint Paul, MN 55108, USA

## Abstract

Genome-wide surveys of transcription depend on gene classifications for the purpose of data interpretation. We propose a new information-theoretical-based method to: assess significance of co-expression within any gene group; quantitatively describe condition-specific gene-class activity; and systematically evaluate conditions in terms of gene-class activity. We applied this technique to describe microarray data tracking *Escherichia coli *transcriptional responses to more than 30 chemical and physiological perturbations. We correlated the nature and breadth of the responses with the nature of perturbation, identified gene group proxies for the perturbation classes and quantitatively compared closely related physiological conditions.

## Abstract 2

A new classification-based framework is presented that allows quantitative description of microarray data in terms of significance of co-expression within any gene group and condition-specific gene class activity.

## Background

The advent of microarray technology has allowed parallel measurements of abundances of thousands of transcripts [1]. The obtained information has been used to describe and understand the transcriptional dynamics in the cell and gene-interaction networks. Such analysis can be reduced to several basic questions: which gene activity makes up a biological response; what are the common characteristics of those genes; and what is the molecular basis of those genes' co-expression? Analysis of multi-dimensional expression data is pivotal to such inferences, and a considerable volume of literature has been published detailing various computational and statistical tools to analyze microarray data. Most of these pattern recognition methods involve classification of profiles of transcript abundances based on proximity or distance, in the expression data space or in a reduced basis space. Such classifications usually yield groups of genes deemed to be co-expressed, and biological interpretations follow to deduce the physiological response of the cells [2-6].

Despite the popularity and wide applicability of these unsupervised techniques, biological significance of those clusters is sometimes difficult to assess because of uncertainties concerning the cluster membership and reproducibility. The clusters or patterns obtained generally consist of a set of genes enriched to various extents for a particular biological function/process/compartment along with genes that cannot be easily co-classified and are forced to fit into a cluster. Under different conditions, these genes may or may not be co-regulated, thus causing the cluster to lose its identity. This observation has spurred the development of condition-specific classification of multiple or large-scale gene expression data. [7-11]. These algorithms largely involve partitioning the expression data into condition-specific groups, in which the expression of genes is most similar across the condition selected for a group. Segal *et al*. [12] demonstrated that expression data can be classified in terms of enriched functional modules and, moreover, these modules can be associated with a regulatory program. Ihmels *et al *[9] proposed an iterative signature algorithm (ISA), in which the entire genome is scanned for groups of genes and conditions that together yield a high threshold score. This algorithm can be seeded with a biologically coherent group of genes, such as genes involved in a pathway, and the iterations will yield a refined module consisting of additional genes that may be associated with the query genes and a set of conditions that the genes are most co-regulated within. In these methods again, it is assumed that a particular program or module is associated with a biological function that is best co-regulated within a set of conditions. However, the ISA method struggles to find coherence within the classified groups, thus running into similar issues that clustering-based algorithms face. Furthermore, these module-based analyses (ISA [9], module maps [10]) only allow for a 'binary' expression program, wherein a group of genes is assumed to be changing direction once during each experiment. Consequently, certain time course experiments (cell-cycle, transient response, and so on) are treated as different conditions since genes change their expression non-monotonously. Importantly, none of these methods account for the background distribution of gene-specific expression, analogous to a statistical null hypothesis. Moreover, all these analyses circumvent the fact that DNA microarray data are noisy. It is desirable that any algorithm proposed to classify gene expression data addresses its sensitivity to background noise, bias and random fluctuations [13]. A systematic study on the effects of data structure, experimental dimensionality and noise levels on the results or reliability of classification techniques employed is yet to be seen.

Classification of unlabeled data based on a training set of query genes is the basis for many supervised classification techniques, like support vector machines [14,15]. In these studies, groups of genes associated with a functional category or a particular transcriptional factor are learned from unclassified data. In an insightful analysis of functional classes in classification of microarray data, Mateos *et al*. [16] observed that only a small percentage of functional classes, derived from the Munich Information Center for Protein Sequences (MIPS), is 'learnable' through machine learning. The reason for this poor performance is attributed to class size (number of genes in the class), class heterogeneity (different members of a class vary their expression in different conditions) and functional interactions between different classes. The authors also observe that groups with low functional heterogeneity and less number of interacting links tend to be better classifiers, and that the behavior of functional classes might be a function of condition.

In this study, we propose a novel method based on a condition-specific entropy reduction of functional groups to determine well-defined physiological responses to diverse experimental treatments. This method does not rely upon any assumptions regarding the dataset, is based on a rigorous statistical formalism, and takes advantage of pre-existing biological classifications to define an experimental result as a set of enriched correlations (and hence, co-expression) for a number of annotated groups of biologically related genes. By measuring how the entropy of a pre-classified group of genes decreases as a function of a condition, we are able to classify transcriptional responses in terms of extent of co-expression of functionally related groups of genes. The expectation is that if genes forming a functional group are genuinely co-regulated under a given condition, the transcriptional profiles of these genes in that condition will be better correlated than in a random assortment of microarray experiments. The group(s) of genes that satisfies this expectation is said to be active, or responsive, in that condition. The significance of entropy reduction of a group-condition is determined by standard statistical criteria, by comparing its activity to permuted background correlation levels of the group. We are, therefore, able to form a coarse, but nonetheless very informative, map of transcriptional responses to various treatments and conditions, and to directly compare two or more groups of genes or conditions. The method is amenable to incorporation of new groups and conditions and flexible enough to allow ready determination of the statistical threshold above which the entropy reduction is termed significant.

## Results

### Characterization of transcriptional responses to experimental stimuli

**Figure 1:**
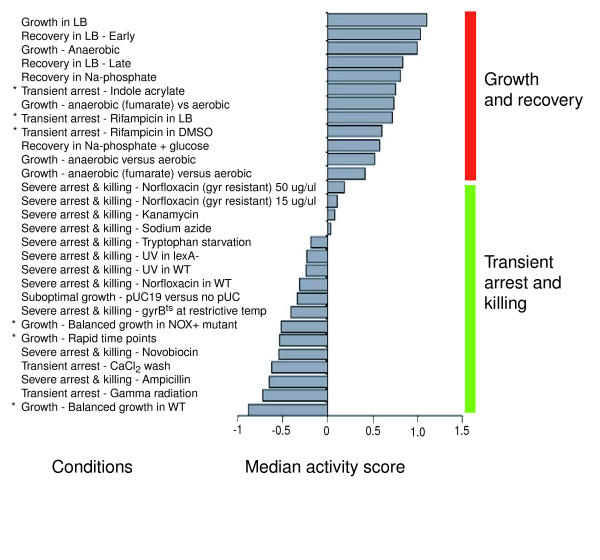
Median scores of experimental conditions classified into 'Growth and recovery' and 'Transient arrest and killing'. Experimental conditions classified into 'growth and recovery' (red vertical bar) and 'transient arrest and killing' (green bar). The conditions are ordered based on their median class activity scores. Conditions of growth and recovery score relatively high on the scale. Low scoring conditions (S_ij _< 0) are those that invoke limited mechanistic responses, and comprise mostly severe arrest and killing type conditions. *Exceptions to the presented experimental classification of conditions. WT, wild type.

Information contained in expression profiles and amplitudes of classified groups of genes is expressed as normalized activity scores (described in Materials and methods). Conditions can be characterized on the basis of either their median class activity or the number and distributions of the high scoring classes. Median class activity for a condition refers to the overall performance of all queried classes in a condition, while the top scoring classes (at least one standard deviation away from the expected scores characterizing transcriptional activity of the class across the conditions and relative to other gene classes) constitute the characteristic transcriptional response for the condition. Low median class activity characterizes conditions that elicit specialized transcriptional responses. Those conditions include, but are not limited to, growth in chemostat at different growth rates, novobiocin, norfloxacin, ampicillin and CaCl_2 _treatment of the wild-type cells, as well as irradiation by UV light or gamma-rays and exposure to temperature upshift. On the other side of the spectrum are conditions in which the transcription of multiple classes of genes is affected (Figure 1). Those are exemplified by aerobic and anaerobic growth in batch cultures, recovery from stationary phase into LB (Luria-Bertani broth) or sodium-phosphate buffer, indole-acrylate and rifampicin treatments

To assess the chief physiological responses in a condition, the classes were sorted for each condition. Conditions that invoke global and wide-ranging responses have higher median class scores and, therefore, have characteristically more classes scoring above zero. High scoring classes in a condition have been further dissected for highly correlated subsets of genes to establish the class expression profile and to infer interesting transcriptional trends from the data (described in Materials and methods). The conditions were analyzed within two general categories - 'Transient arrest and killing' and 'Growth and recovery'.

### Transient arrest and killing

**Figure 2:**
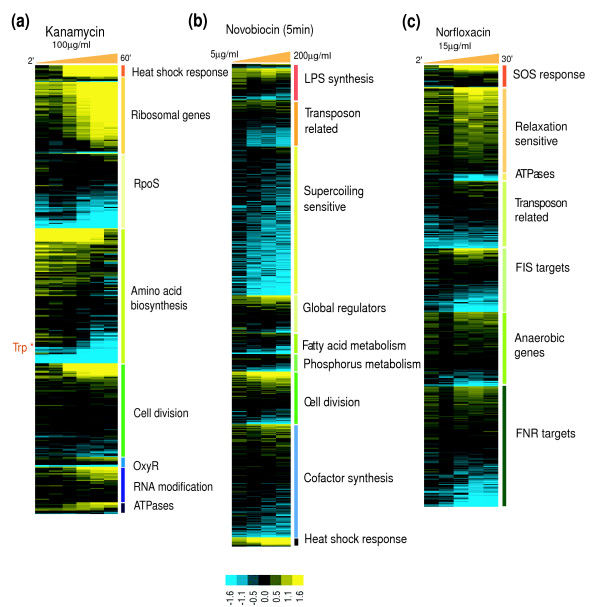
Expression profiles of top-scoring classes for drug treatments. Expression profiles of top-scoring classes (S_ij _> 1) for drug treatments: **(a) **Kanamycin, **(b) **Novobiocin, **(c) **Norfloxacin treatment of the wild-type strain. Classes are sorted from top to bottom in descending order of their scores. A row of pixels corresponds to a single gene expression profile; a blue color indicates relative decrease in transcript abundance, and a yellow color an increase.

In this category, we analyzed and compared transcriptional responses triggered by inhibitors of translation (kanamycin), transcription (rifampicin), replication (norfloxacin and novobiocin), and cell wall synthesis (ampicillin). Individual condition responses are assessed by qualitatively comparing class scores for the condition. In kanamycin treated cells, the response is fairly specific, with heat shock response and ribosomal genes scoring highly among the queried genes. Other groups scoring above the mean in this condition are stress related (RpoS, OxyR), amino acid biosynthesis, cell division related, and genes involved in RNA modification (Figure 2a). Heat shock response in the kanamycin treatment is produced as a result of stalled translation [17]. Both classes expectedly show above the threshold activity scores in this condition. More interestingly, heat shock response is also produced in other conditions of antibiotic and radiation treatment (novobiocin, norfloxacin in gyrase resistant strains, UV irradiation). However, these conditions are characterized by low ribosomal class activity, indicating the uncoupling of heat shock response from ribosomal protein synthesis when translation machinery has not been impacted directly. Another condition in which both classes are highly active is growth in LB, reflective of the fact that heat shock response is also generated when cells are actively translating proteins. The profiles for the two classes are strikingly different in the LB growth condition (and also recovery into LB from the stationary phase), with heat shock response genes being upregulated during the early exponential phase and also during the early stationary phase, while the expression of ribosomal genes decreases with time (Figure S1 in Additional data file 1).

The genes involved in amino acid biosynthesis represent another interesting class in the kanamycin treatment. When we searched this class for correlated profiles of subsets of genes, we observed that genes related to tryptophan biosynthesis (aroM, trpCDE, aroH, tyrA). [18] make up a profile that is anti-correlated with that of the ribosomal genes (Figure 2a).

Novobiocin is a coumarin antibiotic that inhibits ATPase activity of the DNA gyrase [19]. As a result of novobiocin action, DNA gyrase fails to introduce negative supercoils into relaxed or positively supercoiled DNA. When cells are treated with novobiocin, the top scoring classes are lipopolysaccharides (LPS) synthesis, transposon related, supercoiling sensitive genes, global regulators, fatty acid metabolism, phosphorus metabolism, cell division related, cofactor synthesis and heat shock response (Figure 2b). The supercoiling sensitive (SS) genes comprise a group of about 200 genes whose expression is dependent on negative DNA supercoiling [20]. SS genes are significantly downregulated in novobiocin treatment, indicating the inhibition of gyrase function by novobiocin. Additionally, SS genes are upregulated in a concerted manner during anaerobic growth and recovery into LB from stationary phase (data not shown; see scores in Additional data file 3), and they are significantly upregulated by UV irradiation of the wild-type strain (but not in lexA- cells) (Figure S2 in Additional data file 1).

Norfloxacin is a quinolone antibacterial that primarily poisons DNA gyrase and topoisomerase IV, leading to DNA damage. [21]. In wild-type cells, norfloxacin treatment is accompanied by changes in transcriptional activity of DNA damage and recombinational repair (SOS) genes, relaxation sensitive genes (79 genes induced upon DNA relaxation [20]), ATPases, transposon related, targets of FIS, a nucleoid associated transcriptional regulator as well as anaerobic genes and targets of FNR, a regulatory gene for fumarate nitrite, nitrate reductases and hydrogenase (Figure 2c). Thus, it appears that in addition to the transcriptional responses associated with known norfloxacin effects, such as topoisomerase-mediated DNA damage and inhibition of unconstrained supercoiling [22], it also affects genes whose activity is controlled by FIS, a component of a supercoiling-dependent regulatory network and a likely mediator of constrained supercoiling in the cell [23]. In comparison, norfloxacin treatment in gyrase resistant strains affects transcription of genes related to energy metabolism (tricarboxylic acid (TCA) cycle, electron transport, amino acid catabolism) and division (nucleotide synthesis, DNA replication, cell division), apart from the SOS response (Figure S3 in Additional data file 1). This is the only case we are aware of where mutating a drug target leads to a shift, rather than an abrogation, in transcriptional response. This finding is also intriguing because it has been previously observed that secondary mutations rendering quinolone resistance map in the genes of the TCA cycle [24,25]. Furthermore, treatment in resistant strains is characterized by high scores for heat shock response and low scores for relaxation-sensitive genes as the state of DNA supercoiling is not affected in these mutants by the used drug concentrations (data not shown).

Ampicillin treatment induces a response (S_ij _> 1) (see Materials and methods for details of the score calculation) from arginine biosynthesis, sulfur assimilation, amino acid biosynthesis and the LRP (Leucine response protein) regulon. The top scoring classes for other antibiotic treatment conditions are listed in Additional data file 2.

### Growth and recovery

Experiments in this category could be grouped as: anaerobic growth on glucose in M9 media; growth and recovery from stationary phase into LB supplemented with glucose; recovery from stationary phase into sodium phosphate (Na-phosphate) buffer with and without glucose; balanced growth at different growth rates in chemostats (wild type and with NADH oxygenase (NOX^+^) overexpression); recovery in minimal medium following UV and gamma-rays treatment. Most growth experiments are characterized by a large number of classes (>90%) having a positive activity score. Classes that score relatively high in these conditions are related to protein synthesis (ribosomal genes, amino acid biosynthesis), carbon and energy metabolism (TCA, glycolysis, electron acceptors), nutrient uptake and assimilation, global and redox stresses (RpoS, RpoE, polyamine biosynthesis, ArcA, OxyR) and transport proteins (ATP family, Major Facilitator Superfamily, PhosphoEnolPyruvate PhosphoTransferase Systems).

**Figure 3:**
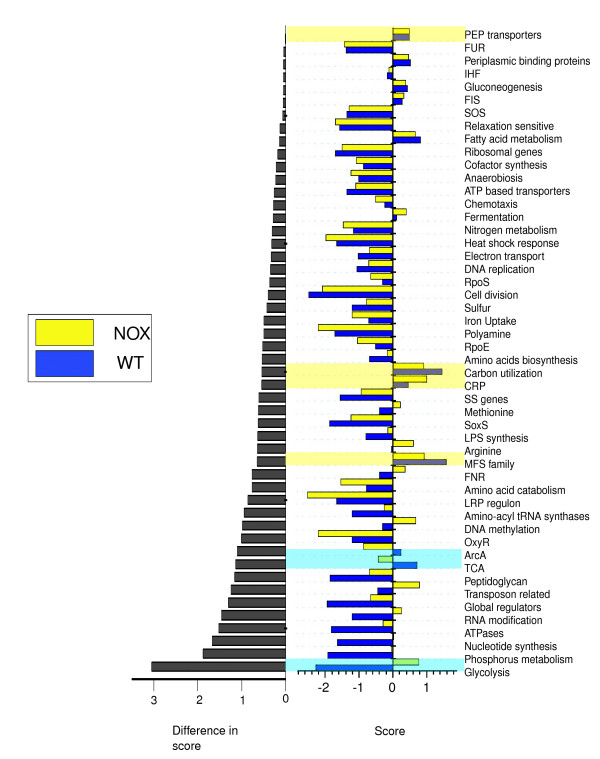
Comparative analysis of class activity scores across balanced growth conditions. Comparison of class activity scores across balanced growth in wild-type (blue) and NOX (yellow) conditions. The classes are sorted according to maximum difference in activities. Both conditions are characterized by relatively few positive class scores - transporters and carbon utilization related classes (highlighted in yellow) - indicating coordinated activity of these genes as a function of condition levels (growth rates). Classes active in the wild type only are highlighted in blue.

When compared to growth experiments in batch conditions, growth in a chemostat under balanced conditions is characterized by lower overall class activity. Also, the top scoring classes in both balanced growth experiments (wild type and NOX^+^) are groups involved in utilization of alternative carbon sources, fatty acid biosynthetic genes and transport proteins involved in uptake of different sugars (Figure 3). The recovery following UV and gamma treatment is accompanied by a narrow range response, primarily composed of genes involved in DNA damage repair and repressed by LexA (SOS genes). Other high-scoring classes in both treatments consisted of DNA replication and supercoiling sensitive genes and regulatory targets of FUR (Ferric uptake regulator). UV treatment is also characterized by the high scoring SoxS regulon, whose genes show upregulation during the treatment, suggesting that cells might also be sensing a superoxide stress. Similarly, gamma radiation can be characterized by activity of the OxyR group and amino acid biosynthesis. As in the norfloxacin treatment, gamma radiation treatment induces a relatively narrow range of responses, as reflected in the low median class activity scores for these conditions (Additional data file 2).

### Class activity across conditions

**Figure 4:**
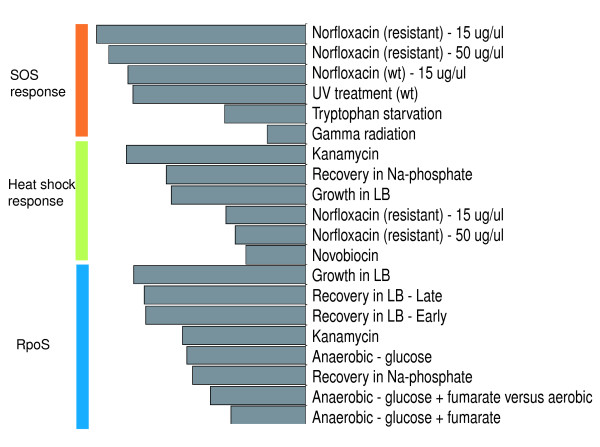
Conditions associated with different stress responses. Top-scoring conditions for three classes: SOS response, heat shock response and RpoS targets. SOS is active in known DNA damaging conditions only (with the exception of tryptophan starvation); RpoS is active in growth conditions (with the exception of the kanamycin treatment), while heat shock response is active in the mixture of conditions.

Apart from individual experiments, it is informative to look at conditions in which classes are co-expressed best. For example, high activity of the SOS class of genes (S_ij _> 1), indicating the sensing of DNA damage by the cells, was observed in a limited number of conditions, including UV and gamma irradiation, norfloxacin (in wild-type and resistant strains) treatment and in tryptophan starvation (Figure 4). In these conditions, the SOS class had a score above 1, while none of the other conditions had a score greater than 0.5 for the class, indicating a clear demarcation in conditions where the response is induced. For the heat shock response class, the top scoring conditions (S_ij _> 1) were treatments of kanamycin, novobiocin, norfloxacin in gyrase resistant strains, growth in LB and recovery in Na-phosphate buffer. While certain drug treatments and exponential growth in rich medium are accompanied by a characteristic heat shock response, it is not clear why this response is induced (transient upregulation) in recovery conditions in LB and Na-phosphate (Figure S1 in Additional data file 1). The less specific stress response class of RpoS is most active in growth and recovery in LB, anaerobic growth, in recovery in Na-phosphate (but not in recovery in glucose added phosphate buffer) and in the kanamycin treatment. When we searched the RpoS class for a subset of highly correlated genes, a group of nine genes (aidB, cbpA, osmY, poxB, dps, hdeA, hdeB, xasA, gadA, gadB, adhE) was found to be significantly correlated (median correlation >0.6) across all conditions tested. The profile of this subgroup during different growth and recovery conditions (Figure S4 in Additional data file 1) indicates that these particular genes are downregulated whenever cells are supplied with abundant nutrients and exposed to kanamycin treatment, and are upregulated whenever cells approach the stationary growth phase.

### Comparison of conditions

Class scores can be compared for different conditions and it can be particularly revealing in comparisons where conditions are similar to each other. Comparisons can be made by assessing the difference in class scores in two conditions, or by grouping together conditions, which are expected to elicit phenotypically similar responses. For example, we can compare conditions of recovery into LB at an early (OD 0.5) or later (OD 1.0) stage. The recovery at higher density is characterized by differential activities of amino acid catabolism, sulfur assimilation, PEP based transporters, phosphorus metabolism, FNR, fermentation, OxyR, SoxS, gluconeogenesis, FUR and ArcA, indicating that cells are undergoing the onset of global nutrient limitation along with redox imbalance (Figure S5 in Additional data file 1). The early recovery condition is characterized by cell wall synthesis (RpoE, LPS synthesis), energy generation (ATPases), supercoiling state related classes (FIS, IHF (Integration Host Factor), relaxation-sensitive), ribosomal genes, amino acid and nucleotide biosynthesis and nitrogen assimilation. Thus, cells early in the growth stage coordinate their regulation towards growth and division, whereas at later points cells encounter nutrient starvation and redox related stresses. Furthermore, recovery-stage dependent induction of RpoS, anaerobic genes, nucleotide synthesis genes and ribosomal genes indicate that the starvation response is fairly independent of the culture's age and history.

Similarly, comparison between the wild-type and NOX^+ ^mutant in balanced growth conditions revealed that TCA and ArcA classes are more active in the wild type, while overexpression of NADH oxygenase (NOX^+^) causes activation of glycolysis, which is the largest difference in the two conditions (Figure 3, highlighted in blue). NOX (encoded by the NADH oxygenase gene from *Streptococcus pneumoniae*) acts as a NADH sink to regenerate the oxidative potential of NAD^+^, thus allowing glucose to be completely metabolized in the cell and relieving the repression of ArcA two-component system (GN Vemuri, DS, ABK, unpublished data). Commonly activated classes in both conditions include the PEP and MFS family of transporters and carbon utilization related genes (highlighted in yellow).

For group comparisons, conditions are classified into three meta-groups based on their phenotypical responses, and classes are sorted for their median activity in the conditions constituting the group. Unlike pairwise comparison of conditions, top scoring classes in a group of conditions constitutes a common 'signature' response for that group. The first group consists of growth and recovery conditions (growth in LB, early and late recovery in LB, recovery in sodium phosphate buffer and glucose-supplemented sodium phosphate buffer; Figure S6 in Additional data file 1). This group is characterized by high activity scores (in decreasing order) for amino acid catabolism, arginine biosynthesis, nitrogen metabolism, RpoS, RNA modification, polyamine synthesis, LRP regulon, nucleotide synthesis, amino acid biosynthesis, PEP transporters, chemotaxis, FIS targets, iron uptake, relaxation sensitive, ribosomal genes and ATPases. Two of the least scoring classes for this group are CRP (cAMP receptor protein) and carbon utilization, with the exception of recovery experiments in sodium phosphate and glucose-supplemented sodium phosphate, indicating the lack of carbon stress in the growing cells. Arginine biosynthesis genes and the RpoS subgroup mentioned in the previous section have a role in acid resistance of cells at the onset of the stationary phase [26]. Comparison of recovery profiles under different conditions (early or late, in buffer with or without glucose) shows interesting trends. Ribosomal genes, RNA modification genes, polyamine synthesis and ATPases are expressed as a strong function of growth conditions and energetic state of the cell. Amino acid biosynthetic genes, with the exception of methionine, glutamine and tryptophan synthesis genes, are repressed in all conditions

The second group consists of treatments by drugs whose modes of action are not known to damage DNA. This group includes conditions of sodium azide, ampicillin, indole acrylate and kanamycin treatments, and it is characterized by high scores for amino acid biosynthesis, arginine synthesis, LRP regulon, peptidoglycan, sulfur assimilation OxyR, nucleotide synthesis and heat shock response (Figure S7 in Additional data file 1). The third group includes DNA damaging conditions of norfloxacin treatment, UV radiation (in wild-type and lexA^- ^mutant), gamma radiation and novobiocin treatment. Not surprisingly, SOS response is by far the top scoring class in this group (with the notable exception of novobiocin treatment and UV treatment in lexA-), followed by heat shock response, cell division genes, DNA replication and supercoiling sensitive genes (Figure S2 in Additional data file 1).

### Comparison with other classification techniques

**Figure 5:**
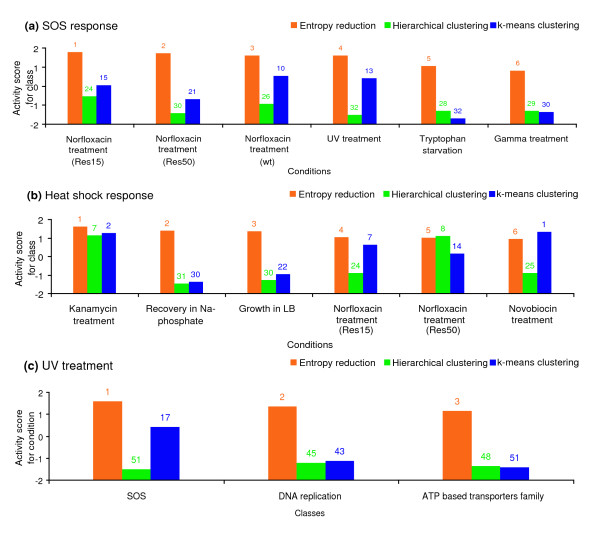
Comparison of the entropy reduction method with standard clustering techniques. **(a) **Normalized activity scores for SOS response. **(b) **Normalized activity scores for heat shock response class. The scores from entropy reduction (orange bar) and clustering (k-means (blue), *k *= 10, and hierarchical (green)) methods are shown. The conditions on the ordinate are top scoring conditions sorted by scores obtained from the entropy method. The ranks for the class for each condition and in each method are listed on top of the respective bars **(c) **Normalized activity scores for classes in UV treatment condition obtained from entropy reduction and clustering methods; classes are sorted by activity scores from the entropy method. The ranks for each class in the condition and in each method are listed on top of the respective bars.

To evaluate the utility of the entropy reduction analysis, we compared the performance of the proposed method with standard unsupervised learning methods [27], such as k-means and hierarchical clustering, and with a more recent technique known as the signature algorithm (SA) [28]. For clustering, we devised a comparable metric (described in Materials and methods) to score the activity of each class (condition) learned from a particular clustering result for a condition (class). For the purposes of illustration, we limited our comparison here to the classes and conditions, SOS and heat shock responses and UV treatment, whose underlying physiology is well understood, thus providing us with a good set of biological expectations. We compared the scores obtained from clustering and the entropy-reduction method for the SOS and heat shock classes of genes, which are expected to produce transcriptional responses in the conditions of DNA damage and growth perturbations, respectively. The comparison revealed that the conditions that are known to cause DNA damage (among all of the tested conditions, five treatments have been specifically set up to elicit this type of response) score consistently on top of the other conditions and higher than they score based on the clustering solutions (Figure 5a). Similar results have been obtained with the heat shock response genes (Figure 5b). Thus, despite a strong expectation that expression of the SOS and heat shock genes should be affected by several conditions, clustering failed to identify these conditions within the dataset. For individual conditions, the entropy-reduction based method is more successful than clustering in identifying top scoring classes that constitute known biological responses to a condition. This is illustrated by a comparative application of the methods to a condition of UV irradiation (Figure 5c). The comparison demonstrated that, unlike in the entropy reduction method, neither the SOS nor DNA metabolism class of genes score high in clustering methods, contrary to the prior biological expectation. Furthermore, classes that are deemed to be significantly different by clustering tend to have lower amplitudes (data not shown), thus reflecting the importance of using both amplitude and profile features to gauge activity of a class.

Next, we compared our method with the SA, a technique that relies on amplitude of expression to refine a seeded group of genes [28]. SA also identifies arrays (that is, a single time point in a condition) in which the group is most activated. By definition, our method differs from the SA: unlike the SA method, our technique maintains the integrity of classes and conditions, scores classes across an entire spectrum of conditions and conditions across all the classes, and the scores are a function of the amplitude, correlation and background expression of the dataset. To compare the performance of the SA with our method, we examined two criteria: how well a particular class is refined by iterating the algorithm; and which conditions are over-represented in the top scoring arrays for a class in SA after the above iterations. Some classes (for example, DNA replication, RNA modification) produced empty sets after iteration, indicating that some classes need to be analyzed as a whole, which cannot be done by clustering or SA. A list of illustrative examples of classes that remained stable is provided in Additional data file 4. The entropy reduction method retained a class subset that is at least equal to that retained by SA for most classes, and in some cases (for example, ribosomal genes, DNA replication, RNA modification, SOS response), it was much higher. Moreover, while SA captures most conditions that our method identifies as most active, it misses out on some biologically relevant examples. Such examples include kanamycin treatment for ribosomal genes (Figure 2a), novobiocin and norfloxacin treatments for heat shock response and recovery in sodium-phosphate buffer for the RpoS group of genes. Furthermore, given available biological evidence, some conditions deemed as differentially affecting certain classes of genes appear to be erroneously classified by the SA. The most striking among them is the classification of sodium azide treatment as the highest scoring SOS specific condition: neither the available experimental data (not shown) nor close examination of the transcriptional patterns of the SOS genes in the condition warrants such an inference. Additionally, in this version of the algorithm, seeding arrays (or conditions) to identify top scoring genes (and hence classes) to identify top responses in specific treatments is not possible, something that can readily be achieved by our technique.

Conclusions from comparisons between these techniques have so far been based on biological expectations, which may prove to be wrong. To test the different methods in an unbiased manner, we generated simulated datasets from the original data, in which a particular gene class was spiked with known profiles in certain conditions. These profiles and their amplitudes represent typical time-series profiles observed in microarray data (for example, late upregulation, early upregulation followed by downregulation, periodic profile and so on). The entropy-reduction method identified exclusively the spiked conditions (score >1) in several randomizations of the background conditions. In comparison, both clustering methods performed poorly, with a false positive and false negative rate of about 50%. The SA performed consistently well in identifying a subset of profiles (three out of seven profiles tested), but it did not identify the remaining profiles in which response was generated only for a part of the time course or periodically, and also in the case in which two subgroups in the same class were anti-correlated (this type of response is expected when a regulator has a dual role of repressor and activator) (Figure S8 in Additional data file 1). Considering this evidence, the entropy-reduction method, in addition to being uniquely suited for describing responses of pre-defined sets of genes in a context of available data without washing out the identity of a set (condition), proves to be more versatile and reliable in classifying non-binary or heterogeneous responses than clustering or signature algorithm.

## Discussion

One of the motivations for doing genome-wide analysis of transcription is to be able to predict the transient state of the cell based on the activity of genes. Ideally one would like to be able to establish a correspondence between a condition, environmental or genetic, and a transcriptional state of the cell; for example, in the simplest of cases, if a gene X changes its activity, it is likely that cells have been subjected to a perturbation Y. While surveying a multitude of controlled conditions for the sake of interpreting the uncontrolled ones may not be practical, in principle it should be possible to obtain a representative sample of conditions that would allow us to: describe individual surveyed condition(s) in terms of gene activity; and present gene activity as a molecular proxy of a particular condition(s). Towards this goal, we obtained and analyzed expression data for more than 3,600 genes in the genome of *E. coli *in more than 30 conditions.

Our analysis is predicated on the notion that rationalization of a transcriptional response is possible only in terms of the already available or emergent information about the groups of genes. The current study took advantage of the breadth of available information about the physiology of *E. coli *bacteria. We used functional and regulatory classifications of genes and their products to evaluate the transcriptional activity within and across groups of related genes. We were also able to describe the examined conditions in terms of transcriptional activity of gene families. The choice to analyze transcriptional responses in the classified groups of genes was dictated by the following. First, given a large number of surveyed genes and a relatively small number of responses, the transcriptional behavior of a group of related genes, where relatedness can be defined by various biological criteria, is likely to be more informative than that of an individual gene. Second, transcriptional patterns obtained by either supervised or unsupervised techniques are being widely interpreted in the context of the already available information about the genes whose respective classes are more represented in the pattern [29]. Such an approach implies a certain degree of co-regulation within the families of genes that have been used to derive the biological meaning of discovered patterns. This assumption about co-regulation has never been explicitly tested. Thus, the third reason, evaluating the degree and homogeneity of co-regulation within the annotated gene families, is of considerable practical and biological interest. Furthermore, any hypothesis regarding a group of unrelated genes, for example, connected pathways, can be tested simply by querying that group in this analysis. Any new condition can likewise be queried for its characteristic response profile from the existing classes.

In this study, we have proposed a novel method for assessing condition-specific co-regulation of pre-classified functional groups based on reduction in Shannon entropy for a group of genes. Previously, some biological studies have used entropy to develop classifiers for microarray data, identify biases and patterns in protein and DNA sequences and to predict drug targets [30-34]. Here, the entropy concept is used to assess the degree of coherence in the expression pattern of functionally related genes in a given condition. This coherence is hypothesized to be a systematic result of class and condition related trends, and this hypothesis is verified or rejected by randomization of classes and conditions. This degree of coherence allows for description and comparison of class-condition behavior on a continuous information scale. By identifying functional classes that show a significant degree of co-expression, large-scale microarray data can now be meaningfully characterized, without relying on assumptions about underlying structure of the data.

The scope and number of surveyed conditions also allowed us to determine whether the observed changes in expression are condition specific and whether the conditions themselves were distinct enough to be characterized by a specialized transcriptional response. The approach proposed in the current study has at least two advantages compared with other methods, which analyzed condition-specific transcriptional patterns. [9,10]. First, condition-specific responses were quantified using a composite metric reflecting both the amplitude of a transcriptional response as well as the information content of a transcriptional profile. Second, the distribution of transcript abundances across all examined conditions was used to assess the background information in transcriptional profiles for a specific condition. The difference between a condition-specific profile and the background activity allows for a rather natural and straightforward way of describing the relative activity of a group of genes. Third, the activity score does not rely heavily on the classification accuracy on the whole, since enhanced correlations in class subsets often 'carry' the class, regardless of the lack of correlation in the remaining genes.

By applying this method to a set of experimental conditions, we were able to validate several beliefs regarding physiological responses to certain stimuli, as well as to discover new trends. For example, cells under normal growth conditions or recovering from the stationary phase are able to co-ordinate genome-wide functional activities, whereas cells under severe stress are significantly less capable of doing so. Cells growing at different balanced growth rates adjust only a part of their metabolic activities to cope with different doubling efficiencies. Drug treatments that are known to affect DNA integrity produce responses dominated by groups of genes involved in DNA metabolism (SOS response and DNA replication). Under conditions of nutrient starvation or stationary phase, cells activate genes related to general stress response, nitrogen limitation and acid resistance. Classes were used as molecular proxies to partition the condition space - SOS (DNA damage versus no damage) and RpoS (growth versus non-growth). Condition-specific correlational links were discovered between functional classes, for example, ribosomal genes correlate with heat shock genes conditionally. Overall, this approach provides a unique and elegant tool for generating the blueprint of transcriptional response to external stimuli. It also provides a platform for further investigations by using significantly co-expressed classes and their subsets as candidates for machine learning and supervised classification.

Modular organizations of transcriptional circuits. [35,36], as well as apparent re-tuning of transcriptional regulation of paralogous genes in mutant backgrounds [37], suggest a certain degree of flexibility in cellular transcriptional programs. While intuitively appealing [38], such flexibility is not fully compatible with the notion of rigidly structured transcriptional modules and regulons. By assessing transcriptional activity of pre-assigned groups of genes we could see that transcriptional activity of the genome can be described through a contribution of multiple functional groups of genes on an essentially continuous information scale. Such a 'continuum' of transcriptional activity across genes in a genome may serve as the basis for inherent flexibility of transcriptional programs. Whereas it may limit the usefulness of genome-wide monitoring of gene expression for screening purposes, it likely offers a more adequate representation of the biology of the system.

## Materials and methods

### Overview of experimental conditions

All experiments were carried out using the MG1655 genetic background from American Type Culture Collection 47076. Relative transcript abundances were measured under conditions of normal growth, sub-optimal growth, transient arrest and recovery and in severe arrest and killing. The following experimental conditions were tested (a detailed summary of comparisons is available in Additional data file 8).

#### Normal growth

During 'normal growth', we tested: the growth curve under anaerobiosis in M9 salts supplemented with 0.2% glucose with or without fumarate as an electron acceptor (24 array-hybridizations, 6 time point comparisons with and without fumarate against two different common references; details of labeling and references are presented in Additional data file 7); the growth curve under aerobic conditions in LB supplemented with 0.2% glucose (11 array-hybridizations, 11 time points along the curve compared to a common reference); recovery of cells from the 24 hours old stationary LB culture into LB + 0.2% glucose at two different inoculum densities (14 array-hybridizations, 7 comparisons against common reference each); recovery of the cells from the 24 hours old stationary LB culture into Na-phosphate buffer, pH 7.5, at an inoculum OD_600 _of approximately 0.5; and recovery into Na-Pi buffer supplemented with 0.2% glucose.

#### Sub-optimal growth

During 'sub-optimal growth', we tested: transient heat-shock treatment (four time points); indole acrylate (IAA) mild starvation at two concentrations of IAA (four time points each); the limited growth curve of the cells harboring pUC19 (five time points); and UV-untreated controls of the wild-type and lexA^- ^cells that were handled similarly to the experimental sample but not treated with the UV light [39] (four time points).

#### Transient arrest

During 'transient arrest', we tested: UV treatment in the wild type (five time points); gamma-ray treatment in the wild type (five time points); Norfloxacin treatment in *gyrA*^r^*parC*^r ^at two different sub-lethal concentrations [20] (ten time points); early stationary cells in LB (six time points); 0.1 M CaCl_2 _treatment in the cold (seven time points); and 0.5% DMSO treatment (two time points).

#### Severe arrest and killing

During 'severe arrest and killing', we tested: treatment with 0.01 M sodium azide (four time points); tryptophan starvation in the auxotrophic strain (three time points); UV treatment of the SOS-uninducible lexA3 mutant (five time points); treatment of wild-type *E. coli *with Norfloxacin at lethal concentrations (five time points); treatment of wild-type *E. coli *with different bactericidal concentrations of Novobiocin (four comparisons after 5 minutes of treatment); shift of the *gyrB*^Ts ^to restrictive temperature (four time points); Rifampicin treatment (500 ug/ml) in LB (five time points) and in M9 + 0.2% glucose (seven time points) [40]; Ampicilin treatment (100 ug/ml) in M9 + 0.2% glucose (six time points); and Kanamycin treatment (100 ug/ml) in M9 + 0.2% glucose (six time points).

### General microarray procedures

We amplified 4,290 *E. coli *open reading frames (96.4% average success rate) using primer pairs from Sigma Genosys (St Louis, MO, USA). EtOH precipitated amplification products were printed on glass surfaces to produce whole-genome DNA microarrays using an in-house 16-tip robotic spotter as described in [41]. Following a print (the data presented in this communication were collected on slides from eight different prints) slides were post-processed as described in [41] and stored in a dark dry environment until hybridization. Total RNA extraction, RNA labeling via direct Cy-dye incorporation into cDNA and array washing were performed as described elsewhere [42]. A 16-bit TIF image was acquired using a GenePix scanner (Axon Instruments, Molecular Devices, Sunnyvale, CA) and analyzed using GenePix software. Raw data of previously published experiments, including UV, rifampicin and norfloxacin treatments, and tryptophan starvation by indole acrylate, have been deposited in the Stanford Microarray Database. [43].

### Data preparation

Raw intensities in individual fluorescence channels were extracted. In the presented analysis, total florescence intensities were used to calculate normalized ratios. All spot-specific ratios were normalized assuming the equality of intensities in both fluorescence channels. Background subtracted ratios were tested on the sub-set of pre-existing groups of genes, such as documented operons and the tightly controlled SOS and tryptophan regulon, and it has been determined that background subtraction increases the scatter in corresponding distributions of correlation coefficients. The final analysis included 3,607 genes. Apart from 155 genes whose amplification products could not be identified unambiguously, genes were filtered out on the basis of inconsistent hybridization results across all 240 arrays; 144 genes were filtered out as their corresponding array elements were flagged, manually or automatically, in 210 out of 219 arrays. Remaining 'poor quality' genes were removed from consideration following the analysis of distributions of spot regression coefficients, intensities and diameters. Of the filtered out genes, 77% encode hypothetical proteins.

The experimental dataset consists of log ratio intensity values for *G E. coli *genes measured in *M *cDNA microarray hybridizations. The *M *arrays correspond to different treatment levels or times in *k *experiments such that:



where *N*_j _refers to the number of arrays in the *j*^th ^experiment. Before collating the data set, values from individual arrays were pre-processed for each experiment, such that means in the arrays are centered on zero. In the case of replicate arrays, average values were considered so that each value with co-ordinates (*g*_*i*_, *r*_nj_) represents the gene expression of gene *g*_i _in unique treatment *r*_nj _(corresponding to the *n*^th ^array in experiment *j*). For the purpose of this analysis, experiments with less than three arrays (or treatments) were not considered, since meaningful correlations can only be derived from a minimum of three data points.

### Query classes

Query classes are groups of genes that are pre-arranged based on some functional relationship. These categories and their corresponding genes were compiled from different publicly accessible *E. coli *databases, including EcoCyc [44], Monica Riley's functional categories at GenProtEC [45] and RegulonDB [46]. The classes chosen for the analysis represent various aspects of cellular physiology and metabolism; selected classes include carbon metabolism (glycolysis, TCA cycle, carbon utilization), DNA metabolism (nucleotide synthesis, DNA replication and degradation, DNA methylation), RNA related (RNA modification), energy metabolism (fermentation related, aerobic respiration, anaerobic respiration, electron transport, oxidative phosphorylation), nutrient uptake and utilization (iron, sulfur, nitrogen, phosphorus), protein synthesis, folding and repair (ribosomal components, amino acid metabolism aminoacyl tRNA synthases, chaperones and proteases), cell division, stress response (SOS response, heat shock response), transport proteins and transcriptional factor targets (RpoS, ArcA, SoxS, OxyR, RpoE, CRP). The size of classes refers to the number of member genes in a class, which typically varied from 10 to 100 genes. A total of 1,642 genes were queried in this analysis, of which 1,104 genes uniquely belonged to a single class, 390 genes belonged to 2 classes and 148 belonged to 3 or more classes. Of the 1,965 genes not included in the classification, 1,466 genes are either unclassified or unknown genes, as described by Riley's classification [45]. The remaining genes either belonged to classes defined purely on the basis of compartmentalization or to loosely defined families of proteins, or to classes with less than five gene members. A list of classes queried along with corresponding genes is given in Additional data file 5. Any set of genes within this range can be queried in this analysis if there is a hypothesis regarding their co-expression. Examples of such classes can include stable clusters obtained from clustering of individual or meta-datasets, or genes belonging to one or related pathways of interest, or genes having a common upstream sequence motif. The choice of query classes could depend on the nature of the experiment and the prior expectations regarding the outcome.

### Shannon entropy

Entropy in thermodynamic terms refers to the degree of disorder in the system. Claude Shannon [47] defined the concept of entropy *H *in information theory as the degree of uncertainty associated with an information source (equation 1):



where *L *stands for the number of states and *p*_i _corresponds to the probability of occurrence in state *i*. An entropy value of 0 stands for a state of high probability and that of 1 corresponds to a highly disordered state with high uncertainty and the state that needs the most amount of information to describe it. The general idea was applied by Alter *et al*. [4] to describe the information contained in the principal eigenvectors obtained by singular-value decomposition (SVD) of a microarray data set. A brief description of the SVD procedure is given in Additional data file 6. Highly ordered and noiseless datasets, with 1 or 2 dominant patterns of behavior, have low entropy, whereas noisy and randomly behaving genes constitute a high-entropy dataset. The concept of entropy has also been applied elsewhere in microarray data analysis to recursively develop a feature-rich training set for classification [30,48] and to validate clustering methods [29].

**Figure 6:**
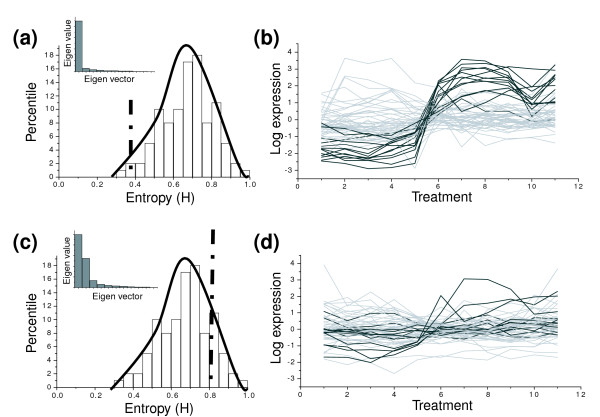
Distributions of entropy values for an active and randomized class-condition. **(a) **Distribution of randomized entropies for an 'active' class-condition pair. The actual entropy for the class is denoted by a vertical line. The percentile counts for the class correspond to the area of the distribution to the right of the dotted line. **(b) **Expression profiles of an active class-condition. The darker gray lines indicate a highly correlated subset in the group. For an 'active' class-condition, a significant portion of the gene members are co-expressed, leading to lower class entropy (see the Scree plot of eigenvalues in the inset). **(c) **Distribution of entropies for the 'inactive' class-condition. **(d) **Expression profiles of the 'inactive' class-condition. Darker lines are relatively few compared to lighter ones, as identified by SVD.

In this method, we evaluated the reduction of entropy within a pre-classified group of genes as a function of condition. Functionally related genes will co-express in certain conditions and not in others. Their enhanced co-expression, or correlation, in a condition will cause the matrix of *g *× *N*_j _log-ratios (*g *∈ *G*) to be decomposed onto fewer eigenvectors, thus causing the Shannon's entropy to be reduced from the universal or background entropy that the group possesses [4]. To get an estimate of the background entropy of the group, the entropy is iteratively calculated for the same group of genes across the same number of arrays picked at random from the dataset (Figure 6). The percentile reduction of entropy for a class is then determined as the number of iterations in which the condition-specific entropy is lower than the randomized group entropy (equation 2):



A high percentile reduction value means that condition-specific class entropy is significantly lower than that of universal (background) class entropy.

The same evaluation is done for a group of genes randomly sampled from the genome for the same condition (equation 3):



Here, a high percentile reduction value means that the condition-specific class entropy is significantly lower than the background condition entropy. High percentile reduction values, for both sets of entropies, implies that genes from a given class are correlated better than expected by chance given available sets of array experiments and expression profiles.

### Amplitude of gene expression

Genes that are highly correlated (and low in entropy) could be those that do not change their activity level at all during an experiment. Also, these could correspond to imputed values in the gene expression dataset. Since genes that do not change their amplitude will be trivially decomposed onto an eigenvector of zero magnitude, such groups will have low entropies and high percentile reduction values. To avoid getting biologically meaningless results, the amplitude of gene expression is considered as the second descriptor of condition-specific class activity. The amplitude of a gene is defined as the sum of squares of expression log-ratios of a gene in the particular condition (equation 4):



where *A*_*ij *_is the total amplitude of class *i *in experiment *j*, *r*_*gn *_is the log-ratio of gene *k *in array *n *and *N*_j _is the number of arrays in experiment *j*. Similar to entropy reduction, amplitude gain for a class is defined as the percentile of condition-specific gain in amplitude for the class-condition over the background (equations 5 and 6):





A combined percentile score is calculated by adding the individual percentile scores for gene-wide and array-wide entropy reduction and amplitude gain (equation 7):



Finally, the scores are normalized to zero mean and a standard deviation of 1 for conditions (equation 8):



where  is the standard deviation of the scores for all classes within a condition.

### Class subset Identification

For classes that show significant entropy reductions (scores above 1), subsets of highly correlated genes were identified. These genes are responsible for maximum reduction in entropy for the class since their profile is represented by a single vector. This is particularly insightful in larger and more heterogeneous classes, such as genes controlled by global regulators that have varied functions and ontologies. The purposes of identifying the subset are: to establish an expression profile for that class; and to collect genes that 'carry the class' in a condition for the purposes of machine learning. Since a class is identified on the basis of its high score, it is expected such a filtered class would be enriched for a single expression profile that can be seen in the gene subset. The expression profile not only allows a visual interpretation of a class's response to a condition, but also indicates whether the significant correlation within a class is supported by a substantial change in gene expression values. The class-subset identification is done by finding genes that correlate maximally with the principal eigenvector of each low-entropy class.

### Comparison with clustering

Using functional annotation to assess physiological responses has its advantages over standard clustering followed by functional interpretations. Clusters are defined to be functionally enriched if a particular class (or classes) is statistically over-represented in the cluster. To analyze which classes are 'learnable' by clustering techniques, we applied the principle of information theory to clustering. We utilize the metric of class-cluster entropy or mutual information (, where *i *refers to *i*^th ^class, *C *refers to clustering result in *j*^th ^condition) to assess which class and how enriched it is in a clustering result [29]. The class-cluster entropy, referred to as mutual information MI for clarity, for a condition reflects how distributed a class is across all resulting clusters in that condition. A lower MI value would indicate that most of the genes in a class are members of one (or few clusters), and a higher entropy value would indicate a wider distribution (equation 9):



where *H*_*ij*_(*A*) indicates the total entropy of a class in a clustering result and *H*_*ij*_(*A*|*C*) indicates the conditional entropy of the class given the clustering result.

Similar to a percentile value defined for class-condition, we define a percentile value for class-cluster by randomizing arrays (equation 10) and clustering each randomized dataset:



where *H*^*AC *^refers to the mutual information of a class in a cluster result. A higher percentile count for a class in a given condition would indicate that: the class is represented in fewer clusters in a condition (enrichment); and the enrichment is specific for a condition over the background for the class. The percentile count is then normalized for a given class over all conditions to define an activity score based on clustering results (equation 11):



The choice of clustering technique was k-means and hierarchical (complete linkage) clustering with Euclidean distance metric over a range of cluster numbers k (6 to 10). This choice was dictated by a previous study that showed that k-means clustering performed better than hierarchical clustering and was comparable to SOM (self-organizing feature map) for a number of datasets, and the optimal cluster number was found to be between 7 and 10 [29].

### Comparison with the signature algorithm

The SA was seeded with classes, and these were refined with a recurrence level of 70% and minimum occurrence of 70% [28] till the set was stable. The number of top scoring arrays for these classes was considered as the maximum of 40 or the number of arrays having scored greater than 50. The enrichment of each condition within these top scoring arrays was calculated from a simple hypergeometric distribution (equation 12):



where *N *is the total number of arrays in the dataset, *m *is the number of top scoring arrays, *K *is the total number of arrays in the condition being tested, and *X *is the event that the top scoring arrays have *j *arrays belonging to the condition being tested. The threshold *p* value for significance was chosen as 0.05. The fraction of genes within a class retained by the entropy method is calculated by considering those genes that have a correlation of at least 0.5 with the principal eigenvector of the expression profile matrix for that class.

### Analysis of simulated class data

A simulated class dataset was generated by randomizing arrays in the expression profiles of SOS genes; 70% of the genes in the class were then spiked with 7 different profiles (including noise) in different conditions (Figure S8 in Additional data file 1). This dataset replaced the original SOS class and was then subject to the entropy method, k-means and hierarchical clustering and the SA. The simulation was repeated several times by generating new randomized class datasets, keeping the spiked profiles constant. Each method was then tested for its ability to identify spiked conditions as active (score >1 or* p* value < 0.05) and to identify the minimum number of false positives, that is, unspiked conditions as active. For SA, the number of top scoring arrays considered was equal to the total number of arrays in spiked conditions. The parameters used for SA were the same as those described above.

The raw data (log expression ratios) used for this study and the description of conditions are available as Additional data files 7 and 8. Part of the data discussed here has been published earlier and is publicly available at the Stanford Microarray Database. [43]; worldwide web links are provided in Additional data file 8. The data introduced in this publication have been deposited in the NCBI Gene Expression Omnibus (GEO) [49] and are accessible through GEO Series accession number GSE4357-GSE4380. The algorithms for comparison of entropies and for subset identification were coded in MATLAB 6.5 [50]. The program for entropy reduction is available as MATLAB code in Additional data file 9, and updated versions will be made available online [51].

## Additional data files

The following additional data are available with the online version of this paper. Additional data file1 contains supplementary figures S1 to S8. Figure S1. Ribosomal and Heat shock genes; Figure S2. Drug (DNA damaging) comparisons; Figure S3. Norfloxacin treatment in resistant strains; Figure S4: Profile of RpoS subgroup in all conditions; Figure S5. Signature classes in LB recovery conditions; Figure S6. Growth conditions comparison; Figure S7. Drug (non-DNA damaging) comparisons; Figure S8. Simulated expression profiles for comparison of methods. Additional data file2 is a table listing the scores of top classes in antibiotic and radiation treatments. Additional data file3 is a table listing the top class scores in growth and recovery conditions. Additional data file 4 is a table listing the comparison of results obtained from entropy reduction and SA. Additional data file 5 is a list of classes and corresponding genes used in the analysis. Additional data file 6 is a description of SVD and the method of entropy calculation. Additional data file 7 is a text file containing the log ratio expression data. Additional data file 8 is a spreadsheet file explaining the conditions used in this analysis and their descriptions. Additional data file 9 is the MATLAB code (EntropyReduce) with sample data files in a compressed (zip) format.

## Supplementary Material

Additional File 1 Figure S1. Ribosomal and Heat shock genes; Figure S2. Drug (DNA damaging) comparisons; Figure S3. Norfloxacin treatment in resistant strains; Figure S4: Profile of RpoS subgroup in all conditions; Figure S5. Signature classes in LB recovery conditions; Figure S6. Growth conditions comparison; Figure S7. Drug (non-DNA damaging) comparisons; Figure S8. Simulated expression profiles for comparison of methods Click here for file

Additional File 2 Scores of top classes in antibiotic and radiation treatments Click here for file

Additional File 3 Top class scores in growth and recovery conditions Click here for file

Additional File 4 Comparison of results obtained from entropy reduction and SA Click here for file

Additional File 5 Classes and corresponding genes used in the analysis Click here for file

Additional File 6 Description of SVD and the method of entropy calculation Click here for file

Additional File 7 Log ratio expression data Click here for file

Additional File 8 Conditions used in this analysis and their descriptions Click here for file

Additional File 9 Sample data files are in a compressed (zip) format Click here for file
