# Supplementary material for: A classification based framework for quantitative description of large-scale microarray data
Source: Genome Biol. 2006 Apr 20;7(4):R32. doi: 10.1186/gb-2006-7-4-r32 (PMC1557986; doi:10.1186/gb-2006-7-4-r32)

SUPPLEMENTARY FIGURE LEGENDS:

Figure S1. Ribosomal and Heat shock genes.
Comparison of profiles of ribosomal and heat shock genes across top scoring conditions. While the two classes are correlated in Kanamycin treatment, their profiles are distinctly uncorrelated in other conditions.

Figure S2. Drug (DNA damaging) comparisons.
Comparison of drug treatments (Norfloxacin treatments in wt and resistant strains, UV treatment in wild type and lexA strain, novobiocin treatment and gamma radiation treatment). The classes were sorted according to median activity in the five conditions. Genes in individual classes were sorted by their weights on the first eigenvector, in the condition of norfloxacin treatment in wt. Global differences across a class can be seen for supercoiling sensitive genes, as well as for SOS and heat shock genes.

Figure S3. Norfloxacin treatment in resistant strains.
Profiles of top scoring classes in norfloxacin treatments (at two dosage levels) in gyrA resistant strains.

Figure S4: Profile of RpoS subgroup in all conditions.
Expression profiles of RpoS subgroup (aidB, cbpA, osmY, poxB, dps, hdeA, hdeB, xasA, gadA, gadB, adhE) show that the genes are significantly correlated (median correlation >0.6) in all conditions. Conditions inducing significant up- and down-regulation of genes are indicated.

Figure S5. Signature classes in LB recovery conditions.
Venn diagram highlighting the top responses in recovery in LB. Common responses in both conditions indicates the ‘signature’ response to growth and recovery while unique responses are determined by the growth stage.

Figure S6. Growth conditions comparision.
Comparison of growth and recovery conditions (in LB, early and late in LB, in phosphate buffer and in glucose added phosphate buffer). The classes were sorted according to the median activity scores in the five conditions. Genes in individual classes were sorted by their weights on first eigenvector, in the condition of LB growth. Patterns of co-activity can be seen in conditions of growth and recovery in LB, but these are lost for most classes in both recovery conditions in phosphate buffer.

Figure S7. Drug (non-DNA damaging) comparisons.
Comparison of drug treatments (Sodium Azide, Ampicillin, Kanamycin and Indole Acrylate (IAA) at two dose levels). The classes were sorted according to median activity in the five conditions. Genes in individual classes were sorted by their weights on first eigenvector, in the condition of Sodium Azide treatment. Predominant classes are related to amino acid metabolism, sulfur assimilation and nucleotide metabolism.

Figure S8. Profiles used for simulation of dataset

A list of profiles with which the simulated class in the dataset was spiked for different conditions. **A**. Downregulation **B.** Late response **C.** Upregulation **D.** Periodic response **E.** Middle stage response **F.** Upregulation and downregulation of subgroups **G.** Significant early response followed by smaller late response. All the profiles were correctly identified by entropy reduction method; however signature algorithm was unable to identify profiles B, D, F and G. Clustering methods were not able to consistently capture all profiles.

Fig. S1: Profiles of ribosomal and heat shock genes

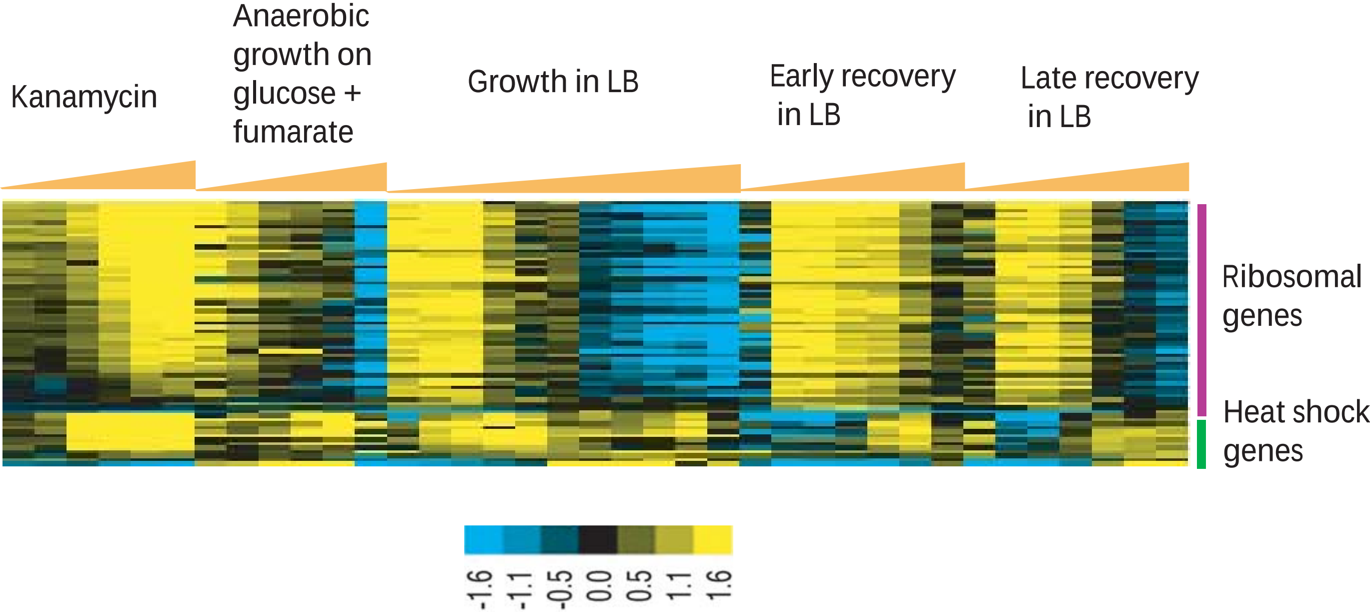

Fig. S2: Drug (DNA damaging) comparisons

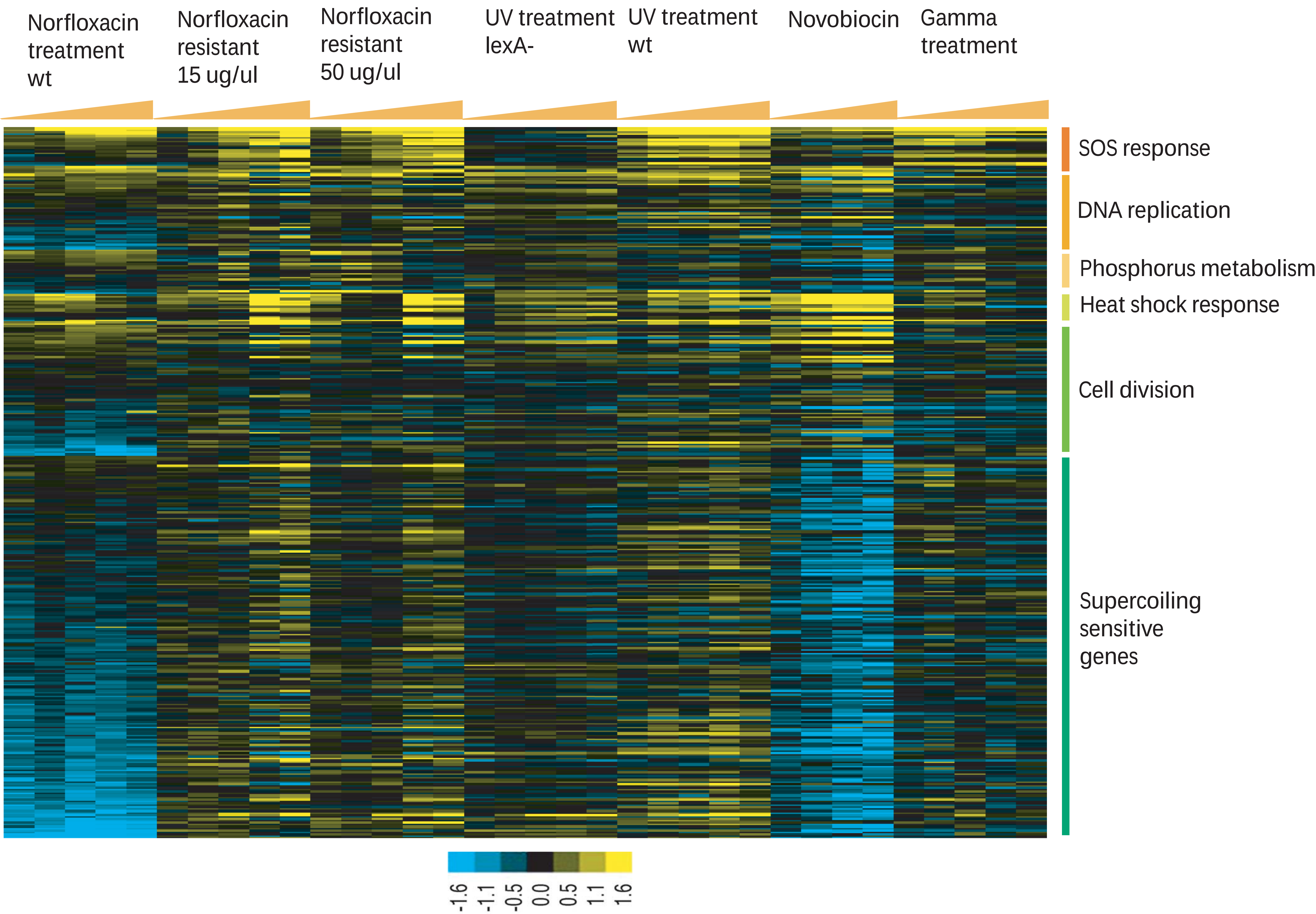

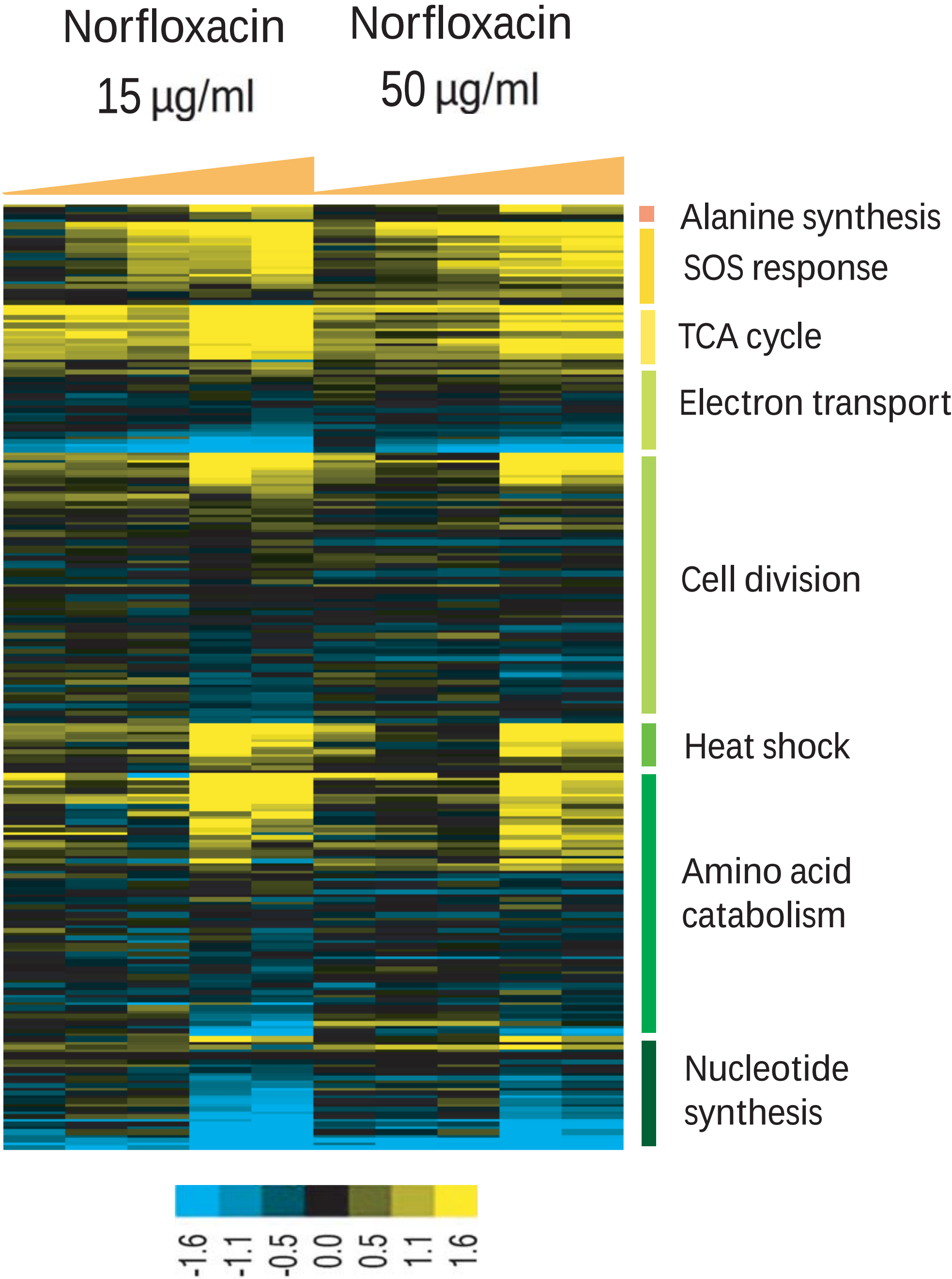

Fig S3: Norfloxacin treatment in resistant strain

Fig. S4

Profile of RpoS subgroup (aidB, cbpA, osmY, poxB, dps, hdeA, hdeB, xasA, gadA, gadB, adhE) in all conditions

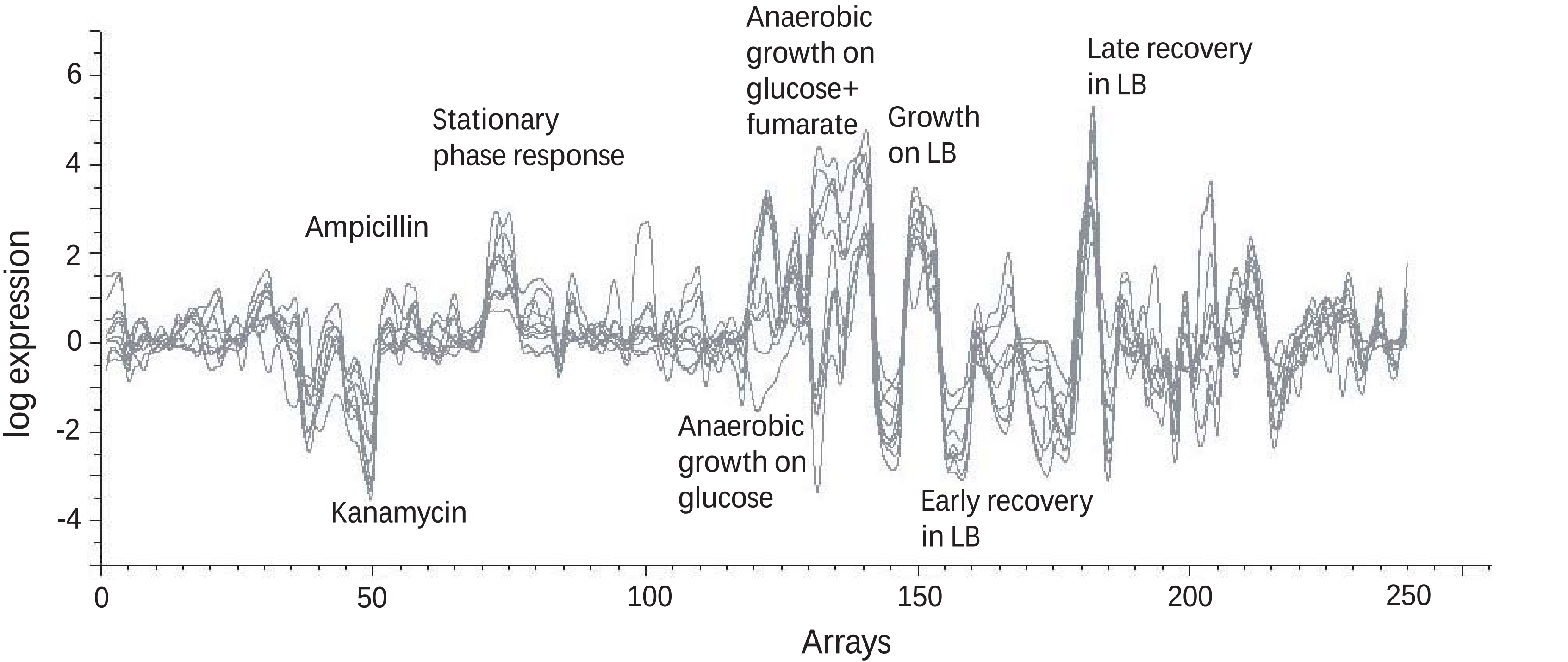

Fig. S5: Recovery conditions in LB

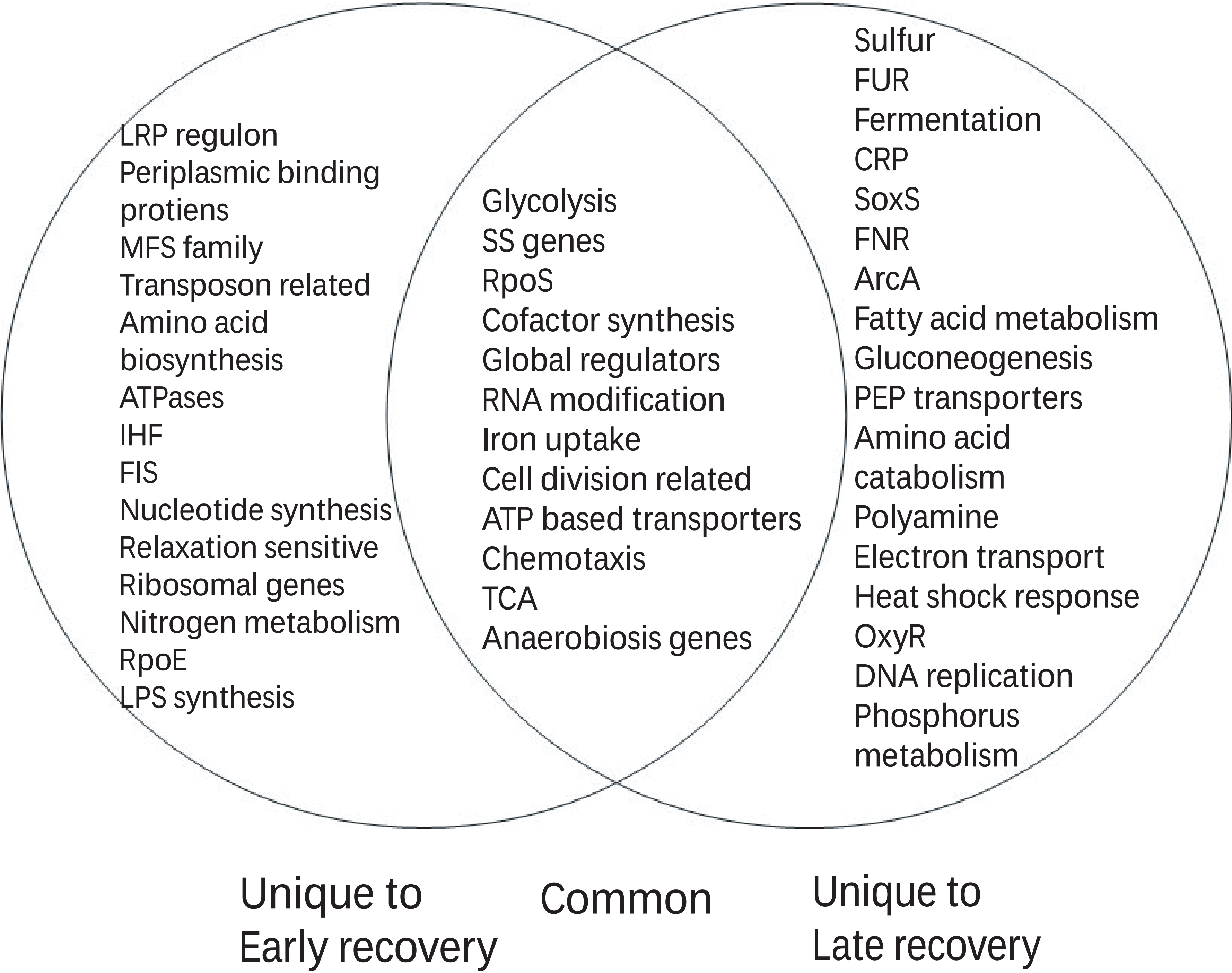

Fig. S6
Growth conditions comparison

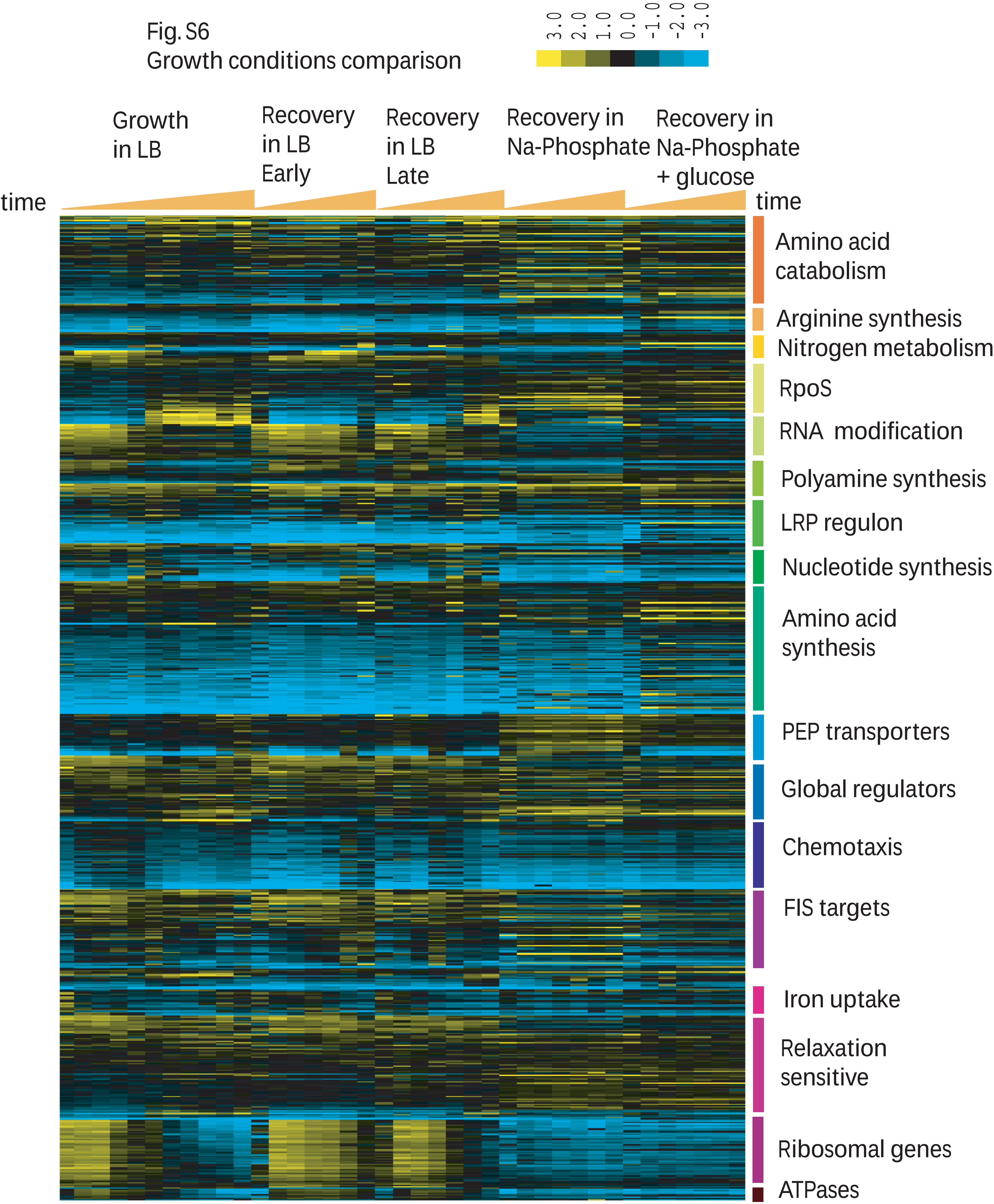

Fig. S7: Drug (non-DNA damaging) comparison

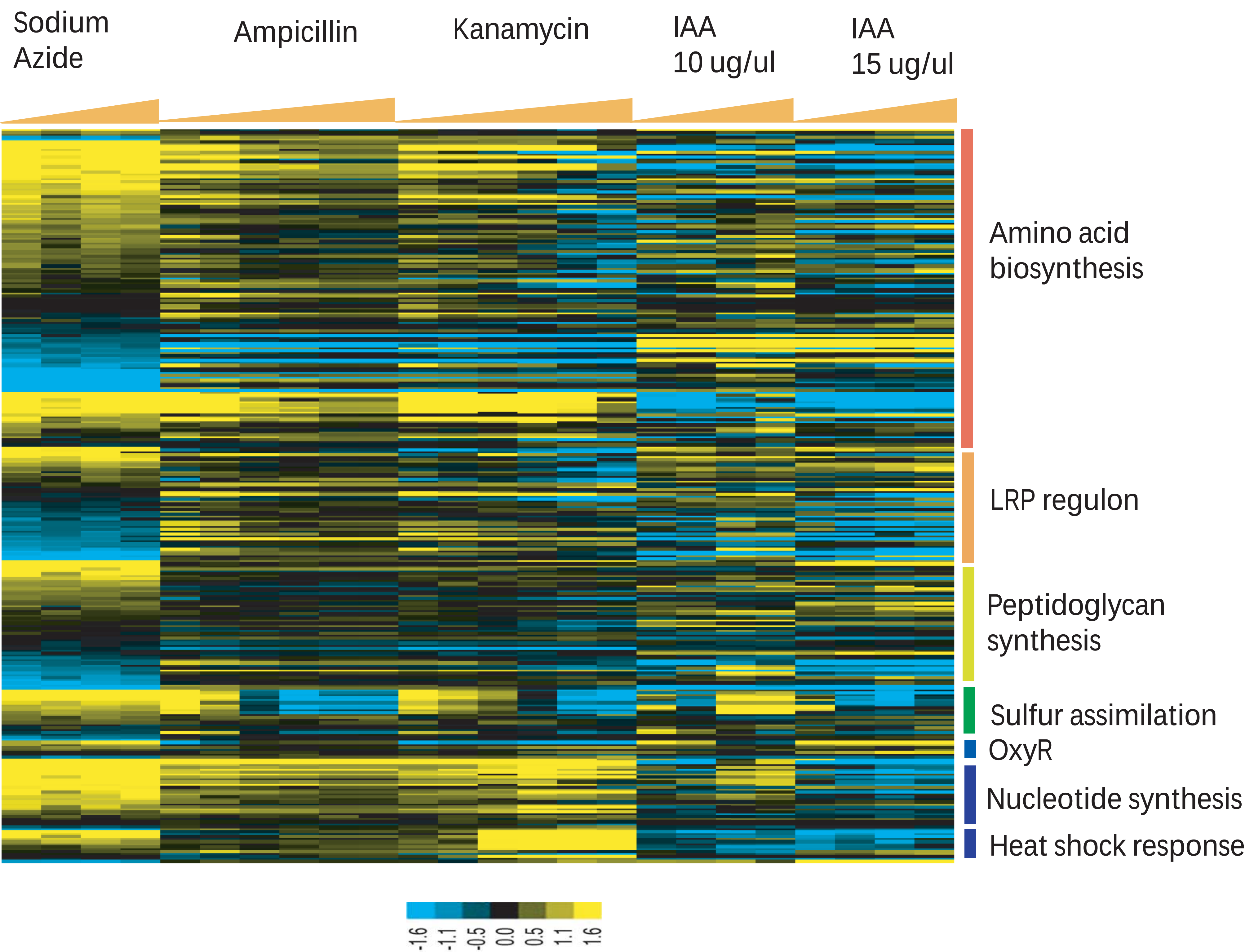

Fig. S8:
Profiles used for simulation of dataset

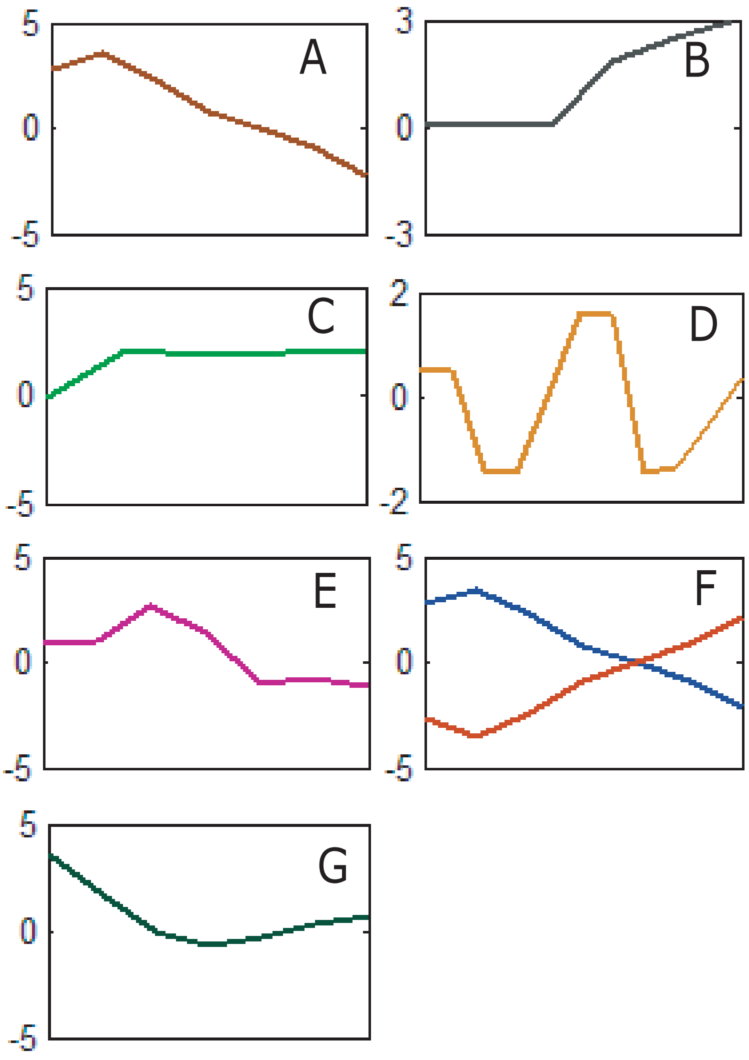

Supplement: Additional File 1 — Figure S1. Ribosomal and Heat shock genes; Figure S2. Drug (DNA damaging) comparisons; Figure S3. Norfloxacin treatment in resistant strains; Figure S4: Profile of RpoS subgroup in all conditions; Figure S5. Signature classes in LB recovery conditions; Figure S6. Growth conditions comparison; Figure S7. Drug (non-DNA damaging) comparisons; Figure S8. Simulated expression profiles for comparison of methods [file gb-2006-7-4-r32-S1.pdf]
