# Supplementary material for: A classification based framework for quantitative description of large-scale microarray data
Source: Genome Biol. 2006 Apr 20;7(4):R32. doi: 10.1186/gb-2006-7-4-r32 (PMC1557986; doi:10.1186/gb-2006-7-4-r32)
Supplement: Additional File 2 — Scores of top classes in antibiotic and radiation treatments [file gb-2006-7-4-r32-S2.pdf]

Supplementary table 1. Drug conditions: Activity scores for queried classes in different drug treatments.

<u>Legend</u>

SA	Sodium Azide 0.01 M
UV lexA-	UV treatment in lexA- mutant
Nf. wt	Norfloxacin treatment in wild type (15 µg/ml)
Nv	Novobiocin treatment (5-200 µg/ml)
Rif LB	Rifampicin in LB medium
Rif M9	Rifampicin in M9 medium
Amp	Ampicillin treatment 100 µg/ml
Kan	Kanamycin treatment 100 µg/ml
Nf. res 15	Norfloxacin treatment in gyrAparC resistant mutant 15 µg/ml
Nf. res 50	Norfloxacin treatment in gyrAparC resistant mutant 50 µg/ml
IAA	Indole acrylate treatment 10 -15 µg/ml

**Class**	**SA**	**UV lexA-**	**Nf. wt**	**Nv**	**Rif LB**	**Rif M9**	**Amp**	**Kan**	**Nf res 15**	**Nf res 50**	**IAA**
TCA	0.33	-0.01	-0.19	-0.15	0.92	0.69	-1.5	0	1.35	0.81	1.77
SOS	0.15	-0.15	1.79	-0.85	-1.19	-1.23	-1.66	-1.18	1.88	1.66	-0.35
Ribosomal genes	-0.05	-1.35	0.78	-1.48	1.69	1.41	-0.02	1.59	-0.28	0.05	-0.55
DNA replication	1.33	0.87	-0.04	0.21	1	0.52	-1.37	0.52	0.43	1.02	0.59
Nucleotide synthesis	0.31	-0.36	-1	-1.11	0.66	1.11	0.73	0.86	1.28	0.58	0.53
Chemotaxis	1.18	-2.26	-0.24	-0.8	-0.05	-1.39	-1.9	-2.26	-0.7	-0.44	1.51
Fermentation	-0.59	-1.06	-1.51	-0.52	-0.95	1.25	-0.76	0.33	0.49	0.44	1.19
Global regulators	-0.9	0.36	-1.38	1.25	1.08	0.72	-0.21	0.3	-0.33	0.57	0.57
Glycolysis	0.7	-0.15	0.1	-0.02	-0.75	0.79	0.74	-0.22	0.42	0.29	1.29
Anaraebiosis	-0.28	-1.72	0.52	-0.39	1.41	0.87	-2.23	-1.34	-0.28	-0.38	-0.02
Periplasmic binding proteins	0.19	0.55	-0.09	-0.16	0.5	1.01	0.95	0.44	-0.03	0.64	0.56
Phosphorus metabolism	0.37	0.09	-1.71	0.83	-0.14	0.28	-1.1	-1.14	0.08	1.04	1.49
CRP	0.14	-1.89	0.39	-0.59	1.54	0.51	-0.58	-1	0.34	1	0.05
Nitrogen metabolism	-0.49	0.23	-0.34	-0.64	-0.06	0.79	0.82	0.71	-1.76	-1.3	1.16
Iron Uptake	-0.17	-1.12	0.37	-1.18	-0.29	0.23	-0.29	0.16	0.03	-1.17	0.94
Methionine	0.71	-0.41	-1.74	-0.28	-1.35	0.46	-0.65	0.47	0.08	-0.15	-0.63
Sulfur	0.88	0.73	-0.95	-0.66	-1.27	1.04	1.41	1.21	1.34	0.88	0.87
Arginine	1.75	-0.38	-0.96	-1.43	-0.08	0.61	1.46	0.12	0.95	-0.21	1.61
Cell division related	0.27	0.35	-0.36	1.38	1.21	0.94	-1.28	1.52	1.27	1.26	0.99
Amino acid catabolism	0.01	-0.14	0.25	-0.99	0.51	0.85	-0.06	0.11	1.32	1.22	0.09
LRP regulon	0.64	0.48	0.55	-0.52	0.88	0.15	0.87	0.72	-0.09	0.23	1.43
Heat shock response	0.44	0.34	0.27	1.28	-0.91	0.03	0.38	1.85	1.38	1.33	0.94
RpoS	-0.18	0.09	-1.32	-1.15	0.73	0.32	0.69	1.61	-1.51	-1.33	-0.31
RpoE	-1.15	-1.29	-1.17	-0.83	1.06	0.66	-0.02	0.48	-1.78	-2.07	1.46
OxyR	-0.48	-1.15	0.07	-0.96	0.56	0.79	0.55	1.45	-0.27	-1.38	1.65
SoxS	0.47	-0.82	0.11	-0.19	0.03	0.43	-0.73	-0.29	-0.16	0.6	0.31
ArcA	-1.47	0.42	0.99	-0.37	1.54	1.41	-1.78	-0.29	1.07	0.91	1.79
Electron transport	-0.91	-0.94	-1.41	-1.67	1.45	1.08	-0.71	-1.75	1.28	0.22	-0.29

**Class**	**SA**	**UV lexA-**	**Nf. wt**	**Nv**	**Rif LB**	**Rif M9**	**Amp**	**Kan**	**Nf res 15**	**Nf res 50**	**IAA**
Peptidoglycan	0.24	0.43	-0.14	-0.66	0.09	-0.26	-0.19	-0.03	-1.19	-1.64	1.72
RNA modification	-0.03	-0.73	-0.88	-0.48	0.73	0.39	-0.69	1.23	0.52	0.8	0.68
Fatty acid metabolism	-0.22	0.48	0	1.2	1.32	0.32	0.09	0.05	-0.7	0.72	-0.56
Polyamine	0.08	0.62	-1.19	-0.4	0.69	0.53	-0.8	0.7	0.86	0.18	0.04
Amino Acids Biosynthesis	1.63	0.08	-2.15	-1.13	0.01	0.91	1.35	1.45	-1.57	-1.25	1.56
MFS family	-0.5	-1.3	-0.27	-0.8	1.28	1.02	-1.32	-0.98	-0.39	-0.17	1.01
ATP Transporters family	0.85	-0.45	-1.74	-1.38	-0.34	1	-0.17	-0.58	-1.68	-0.77	0.49
Cofactor synthesis	-0.5	-0.9	-1.82	0.45	0.69	-0.13	-1.72	-0.51	-0.5	-1.08	0.99
LPS synthesis	0.18	0.16	-0.16	0.96	0.59	0.76	-1.87	-1.3	0.18	-1.41	0.51
Gluconeogenesis	0.38	-0.49	-0.4	-0.63	-0.09	0.53	-0.66	0.12	1	0.51	0.63
ATPases	0.02	-1.38	1.3	-1.86	0.99	0.58	0.61	0.99	-0.34	0.16	0.36
PEP transporters	-0.85	0.43	-1.78	-0.56	1.53	1.3	-0.75	-0.94	-0.65	-1.04	1.09
Transposon related	0.1	-0.53	0.87	1.23	-1.37	-0.53	-2.14	0.1	-1.65	-0.12	-0.37
Carbon utilization	-0.62	0.27	-0.81	-0.64	0.76	-0.01	-1.78	-1.16	-0.01	0.12	0.92
FUR	-0.01	0.6	0.12	0.46	-0.49	1.25	-0.15	0.79	-0.35	-0.67	0.55
FNR	-1.35	-1.55	1.2	-0.62	1.53	1.68	-1.64	-0.36	0.53	0.52	1.83
Alanine	1.88	-1.83	-0.86	-1.2	-0.98	0.1	0.58	0.25	1.55	1.04	1.39
Amino-acyl tRNA synthases	1.37	-0.75	-0.48	-0.9	1.57	1.26	-0.15	0.02	0.1	-0.22	-0.9
SS genes	0.03	0.77	-0.28	1.13	1.65	0.8	-1.14	-0.01	0.24	-0.15	0.85
DNA methylation	-1.67	-0.6	-2.12	1.43	0.57	0.59	-1.58	-1.21	-0.65	-0.43	1.81
FIS genes	-0.21	0.95	1.4	0.54	1.58	0.72	-0.57	0.22	-0.44	-0.41	1.51
IHF	-0.32	-0.36	1.09	-1.53	0.27	1.09	0.62	0.97	0.85	0.82	1.88
Relaxation sensitive	-0.48	-0.94	1.05	0.61	0.61	-0.36	-1.44	0.37	-2.06	-1.17	-0.36
