# Supplementary material for: A classification based framework for quantitative description of large-scale microarray data
Source: Genome Biol. 2006 Apr 20;7(4):R32. doi: 10.1186/gb-2006-7-4-r32 (PMC1557986; doi:10.1186/gb-2006-7-4-r32)
Supplement: Additional File 4 — Comparison of results obtained from entropy reduction and SA [file gb-2006-7-4-r32-S4.pdf]

Table 1: Results of comparison of entropy reduction method with Signature Algorithm.

Class	*Fraction of class retained by SA*	*Significant conditions by SA (p<0.05)*	*Fraction of class retained by entropy reduction*	*Significant conditions by entropy reduction*
Ribosomal genes	58 %	1. Recovery in Na-phosphate<br>2. Anaerobic growth in glucose<br>3. Growth in LB	88%	1. Anaerobic growth in glucose<br>2. Early recovery in LB<br>3. Growth in LB<br>4. Rifampicin treatment<br>5. Kanamycin treatment
SOS response	47 %	1. Sodium azide treatment<br>2. UV treatment<br>3. Gamma treatment<br>4. Tryptophan starvation<br>5-7. Norfloxacin treatment in WT and resistant strains	61 %	1-3. Norfloxacin treatment in wt and resistant strains<br>4. UV treatment<br>5. Tryptophan starvation<br>6. Gamma treatment
Heat shock response	69 %	1. Recovery in Na-phosphate buffer<br>2. Kanamycin treatment<br>3. Recovery in Na-phosphate buffer + glucose	67 %	1. Kanamycin treatment<br>2. Recovery in Na-phosphate buffer<br>3. Growth in LB<br>4-5. Norfloxacin treatments in resistant strain<br>6. Novobiocin treatment
RpoS targets	23 %	1. Early recovery in LB<br>2. Growth in LB<br>3. Late recovery in LB<br>4. Kanamycin treatment	22 %	1. Growth in LB<br>2. Late recovery in LB<br>3. Early recovery in LB<br>4. Kanamycin treatment<br>5. Anaerobic growth in glucose<br>6. Recovery in Na-phosphate buffer
