# Supplementary material for: A classification based framework for quantitative description of large-scale microarray data
Source: Genome Biol. 2006 Apr 20;7(4):R32. doi: 10.1186/gb-2006-7-4-r32 (PMC1557986; doi:10.1186/gb-2006-7-4-r32)
Supplement: Additional File 6 — Description of SVD and the method of entropy calculation [file gb-2006-7-4-r32-S6.pdf]

Calculation of entropy from Singular Value Decomposition of microarray data (according to **Alter et al,** PNAS 2000 (97) 18:10101:10106**)**

Microarray data can be expressed as a matrix of G rows (corresponding to genes) and P columns (arrays). To calculate the entropy of a subset of g X N_j matrix (M), singular value decomposition (also known as Principal Components Analysis) is performed on the centered matrix. SVD is a linear decomposition technique which identifies the full set of eigenvectors of a given non-singular matrix. The eigenvectors can be sorted by their eigenvalues, which assigns weights to the vectors.

$$\sum_{k \times k} = V * S * V^T$$

where $\sum_{k \times k}$ is the covariance matrix of M and k is minimum of (g,N_j). The columns of matrix V are the eigenvectors of the matrix M and S is a matrix which has the corresponding eigenvalues λ_i^2 on the diagonal and zeros elsewhere. (λ_i is the corresponding i^{th} singular value of the matrix M.)

The fractional contribution of each eigenvector to the information can be calculated from its eigenvalues as follows:

$$p_i = \frac{\lambda_i^2}{(\sum_{j=1}^{k} \lambda_i^2)}$$

These contributions of eigenvectors of the matrix M are used to calculate the Shannon's entropy of the matrix:

$$0 \le H = \frac{-1}{\log(L)} \sum_{i=1}^{N_j} p_i \log(p_i) \le 1$$

Where p_i is the fractional contribution of eigenvectors, L is the rank of the matrix and H is Shannon's entropy bounded between (0,1).
