# Supplementary material for: A classification based framework for quantitative description of large-scale microarray data
Source: Genome Biol. 2006 Apr 20;7(4):R32. doi: 10.1186/gb-2006-7-4-r32 (PMC1557986; doi:10.1186/gb-2006-7-4-r32)
Supplement: Additional File 9 — Sample data files are in a compressed (zip) format [file gb-2006-7-4-r32-S9.zip › Matlab code/README.doc]

***EntropyReduce (Version 1)***

A method to assess condition-specific gene-set activity in large scale microarray data.

© 2006 Dipen Sangurdekar, Arkady B. Khodursky; Khodursky Lab at the University of Minnesota, St Paul, MN. Email: sang0036@umn.edu

**Requirements**

This program can be run on MATLAB ([http://www.mathworks.com](http://www.mathworks.com/)) versions 6p5 or higher, having the statistics toolbox. All files provided with the program (especially the .m files) should be unzipped and placed in the same folder. If the folder is not in the Matlab home directory, add the path to the folder using *addpath* function. The program takes about 10 min to run for 500 randomizations on a 2.5+ GHz Pentium 4 CPU with 750 MB RAM to query 30 conditions for 50 classes. For slower computers, running for more than 500 iterations (especially for smaller datasets with fewer than 200 arrays) is not recommended.

This program has been tested for a dataset having at least 10 experiments with each having at least 3 arrays. If the user does not have these many experiments, it is recommended to append his/her data to the sample data file provided to get a better representation of the background. The same applies for classes being queried. The number of genes in each class should equal at least the number of arrays in the smallest experiment/condition submitted. Eg. If one of the experiments contains only 3 arrays, the number of genes in all classes should be at least 3 or more.

**File formats**

The program requires 3 files in tab-delimited text format. Sample files (data.txt, list.txt, classes.txt) are provided with the program.

File 1) **Data file:** This file contains the expression values (log-ratio) of genes in different conditions. The first row should be the header line describing the columns. The first or first two columns are gene information in alphanumerical format. The gene information from the first column will be output in the results file. The gene order and number of genes should be the same as given in data file. There should be no blanks in the data, replace all blanks with either zero or imputed values. Example of a data file (data.txt):

UID	NAME	Condition 1 Array 1	Condition 1 array 2	Condition 1 array 3

B0001	thrL	0.784	0.522	0.528

B0002	thrA	0.215	-0.037	0.005

B0003	thrB	0.193	0.155	0.229

B0004	thrC	0.420	-0.071	0.127

B0006	yaaA	-0.008	0.299	0.172

B0008	talB	2.032	1.656	1.854

B0009	mog	0.571	0.421	0.155

File 2) **Experiment list file:** This file contains the list of experiments (conditions) and the beginning and end array numbers for each in the data file provided. The first column is the condition name in alphanumerical format (no numbers) and the next two columns are array begin and array end coordinates. Do not have header rows. Example list file (list.txt):

Condition 1	1	5

Condition 2	6	11

Condition 3	12	15

File 3) **Class files:** This file contains the gene classification information. Class membership of the list of genes provided with the data file is described as ‘1’ for present in a class or ‘0’ for absent. The first or first two columns are gene information in alphanumerical format. The first row is the header row containing information containing class names. The class file should have the same number of rows as the data file.

Example class file (class.txt)

UID	NAME	Class 1	Class 2	Class 3	Class 4

B0001	thrL	1	0	0	0

B0002	thrA	1	0	0	0

B0003	thrB	1	0	1	0

B0004	thrC	1	0	1	0

B0006	yaaA	0	1	0	0

B0008	talB	0	0	0	0

B0009	mog	0	1	0	0

B0010	yaaH	0	0	0	0

B0012	htgA	0	1	0	0

B0013	yaaI	0	0	1	0

B0014	dnaK	0	0	1	0

B0015	dnaJ	0	0	0	0

**Running the program**

- To run the program, change the MATLAB home directory to the directory where the program is saved, or add the path to the bottom of the current directory using ‘*addpath*’ command. To start the program, type

*EntropyReduce*

in the command window. This will initiate the program with default 100 randomizations of the background. To change the number of iterations to say 500, type

*EntropyReduce(500)*

Iterations above 500 are not recommended for slower computers and small datasets.

[Note: In some examples, input classes may resemble a random group, especially if the class is large or derived not from annotations but from a cluster result. In such a case, normalizing a class percentile score, by the mean and standard deviation of class activity across conditions (as shown in Eq. 8 of the paper) may produce artificially high scores even though the group performs like a random group. An alternative normalization procedure is to normalize by the mean and standard deviation of percentile counts of *all* classes in *all* conditions. To perform this operation, type

*EntropyReduce(B, ‘center’)*

where B is the number of iterations. Note that by default (one or no input arguments), the program normalizes by considering individual class activity as reported in the paper. ]

- Once the program is run, it will prompt the user to input the appropriate files (expression data, classification data and experiment list).

If the data contains an experiment (Test) with fewer than 3 arrays, the following error will be reported

‘ERROR: Number of arrays within condition Test is too low - Exiting program’

and the program will quit.

If the class list contains a class with fewer genes than the minimum number of arrays within any condition, the program will give the following error message and quit

‘ERROR: One or more classes contains fewer genes than the total number of arrays in one or more conditions - Exiting program’

If any conditions contains fewer than 3 arrays, the program will give the following error message and quit

‘ERROR: Number of classes/conditions should be at least 3 - Exiting program’

Error message will also be displayed if the number of genes in data file does not match the number of genes in the class file.

- Upon successful loading of the data, the program will display ‘Done loading data’ and ‘Starting Analysis’

Following the completion the program, the user will be prompted to save the results files in a given path. The result files will be stored in two different formats: 1) An .xls file containing the scores (with classes as rows and conditions as columns) will be stored as a filename prompted by the user. 2) A second MAT file containing the entire data will be automatically dated and stored as file ER-date.MAT in the same folder. This file can subsequently be loaded for further analysis and comparisons.
